# Supplementary material for: Targeting the ceramidase ACER3 attenuates cholestasis in mice by mitigating bile acid overload via unsaturated ceramide-mediated LXRβ signaling transduction
Source: Nat Commun. 2025 Mar 2;16:2112. doi: 10.1038/s41467-025-57330-7 (PMC11873283; doi:10.1038/s41467-025-57330-7)
Supplement: Supplementary file 1 — Supplementary information [file 41467_2025_57330_MOESM1_ESM.pdf]

Supplemental Tables

Table S1. Clinical characteristics of patients in the study cohort.

Variables	Non-CLI	CLI	*P*
Sample size (n)	30	30	
Age (years)	52.13 ± 9.33	59.90 ± 9.11	0.002*
Male (%)	50.00	60.00	0.436
BMI (kg/m^2)	23.46 ± 3.33	22.48 ± 3.37	0.264
HBsAg positive (%)	13.33	23.33	0.317
Cirrhosis (%)	0	16.67	0.020*
Hyperlipidemia (%)	26.67	20.00	0.542
Pathology (%)			< 0.001*
Intrahepatic duct stone	0	36.67	
Cholangiocarcinoma	0	63.33	
Focal nodular hyperplasia	30.00	0	
Hemangioma	70.00	0	
WBC (×10^9/L)	6.60 ± 1.21	7.90 ± 2.41	0.010*
LYM (×10^9/L)	2.24 ± 0.46	1.46 ± 0.75	< 0.001*
NEU (×10^9/L)	3.60 ± 1.24	5.70 ± 2.41	< 0.001*
CRP (mg/L)	1.76 ± 1.56	24.17 ± 17.67	< 0.001*
TBA (μmol/L)	5.69 ± 2.49	98.33 ± 58.13	< 0.001*
TBIL (μmol/L)	10.85 ± 4.34	121.43 ± 94.52	< 0.001*
DBIL (μmol/L)	3.98 ± 2.39	95.06 ± 80.04	< 0.001*
ALT (U/L)	20.57 ± 9.49	177.97 ± 122.50	< 0.001*
AST (U/L)	18.80 ± 4.44	165.10 ± 105.64	< 0.001*
ALP (U/L)	76.20 ± 16.50	255.23 ± 97.15	< 0.001*
APTT (sec.)	26.61 ± 2.32	25.01 ± 3.67	0.049*
PT-INR	0.92 ± 0.07	1.01 ± 0.11	< 0.001*
CR (μmol/L)	67.97 ± 16.78	74.63 ± 31.14	0.306

* Indicates statistical significance, $P < 0.05$. Statistical significances were tested by the unpaired two-sided Student's *t*-test.

WBC, white blood cell count, LYM, lymphocyte count, NEU, neutrophil count, CRP, C reactive protein, TBA, total bile acid, TBIL, total bilirubin, DBIL, direct bilirubin, ALT, alanine aminotransferase, AST, aspartate aminotransferase, ALP, alkaline phosphatase, APTT, activated partial thromboplastin time, PT-INR, prothrombin time-international normalized ratio, CR, creatinine.

Table S2. Clinical characteristics of patients of different genders in the study cohort.

Variables	Non-CLI			CLI		
Gender	Male	Female	*P*	Male	Female	*P*
Sample size (n)	15	15		18	12	
Age (years)	52.33±9.48	51.93±9.50	0.909	56.95±10.04	64.33±5.25	0.027*
BMI (kg/m^2)	23.87±3.08	23.05±3.62	0.513	21.92±3.69	23.33±2.77	0.272
HBsAg positive (n)	2	2	1.000	6	1	0.113
Cirrhosis (n)	0	0	N/A	3	2	1.000
Hyperlipidemia (n)	4	4	1.000	4	2	0.709
Pathology (n)						
Intrahepatic duct stone	0	0		7	5	
Cholangiocarcinoma	0	0	0.046*	11	7	0.879
Focal nodular hyperplasia	7	2		0	0	
Hemangioma	8	13		0	0	
WBC (×10^9/L)	6.95±1.31	6.14±0.95	0.064	7.78±1.89	8.07±3.12	0.777
LYM (×10^9/L)	2.27±0.49	2.24±0.43	0.857	1.68±0.81	1.15±0.55	0.059
NEU (×10^9/L)	3.95±1.39	3.12±0.89	0.063	5.33±1.97	6.27±2.94	0.345
CRP (mg/L)	1.92±1.64	1.60±1.53	0.584	22.75±12.02	16.31±12.37	0.166
TBA (μmol/L)	6.50±2.67	4.88±2.09	0.075	105.50±68.12	87.58±39.06	0.418
TBIL (μmol/L)	11.01±4.37	10.69±4.46	0.841	126.52±96.77	113.81±94.75	0.725
DBIL (μmol/L)	4.46±3.03	3.50±1.46	0.279	97.54±80.46	91.33±82.82	0.839
ALT (U/L)	23.20±11.64	17.93±6.01	0.134	169.17±132.68	191.17±109.72	0.638
AST (U/L)	18.07±4.06	19.53±4.81	0.375	151.44±115.21	185.58±90.25	0.395
ALP (U/L)	77.60±18.33	74.80±14.96	0.650	258.83±105.58	249.83±87.18	0.809
APTT (sec.)	27.17±1.48	26.05±2.88	0.192	26.18±3.00	23.26±4.00	0.030*
PT-INR	0.92±0.07	0.92±0.06	0.848	1.03±0.12	0.99±0.10	0.310
CR (μmol/L)	79.60±14.25	56.33±9.49	<0.001*	81.94±36.80	63.67±15.65	0.117

* Indicates statistical significance, $P < 0.05$. Statistical significances were tested by the unpaired two-sided Student's *t*-test.

WBC, white blood cell count, LYM, lymphocyte count, NEU, neutrophil count, CRP, C reactive protein, TBA, total bile acid, TBIL, total bilirubin, DBIL, direct bilirubin, ALT, alanine aminotransferase, AST, aspartate aminotransferase, ALP, alkaline phosphatase, APTT, activated partial thromboplastin time, PT-INR, prothrombin time-international normalized ratio, CR, creatinine.

Table S3. Grid Scores between human LXRβ and CER(d18:1/18:1) (kcal/mol).

PDB ID	pose	Grid Score	Grid vdW	Grid es	Internal energy
**5i4v**	**1**	**-117.30**	**-108.83**	**-8.48**	**19.35**
6s4t	1	-110.54	-103.86	-6.67	17.23
1pq6-A	1	-105.76	-103.69	-2.07	18.87
	2	-102.77	-88.36	-14.41	20.34
4dk7	1	-92.90	-91.33	-1.57	27.18
4dk8	1	-81.48	-77.88	-3.60	17.17
5kya	1	-77.42	-74.63	-2.79	26.39
	2	-70.93	-69.57	-1.36	44.09
5hjp	1	-77.03	-73.49	-3.54	25.21
	2	-71.75	-70.59	-1.16	25.11
4nqa	1	-74.40	-69.25	-5.15	40.13
	2	-72.93	-69.61	-3.32	46.90
5kyj	1	-73.86	-67.66	-6.20	19.88
	2	-72.20	-66.87	-5.33	23.70
6k9m	1	-70.86	-66.15	-4.71	26.79
	2	-69.62	-64.31	-5.31	21.12
	3	-69.34	-64.14	-5.20	19.76
6jio-A	1	69.22	68.17	1.05	64.07
6s4n	1	-63.69	-63.85	0.17	29.73
	2	-53.49	-53.69	0.19	36.07
	3	-50.54	-48.94	-1.60	52.99
6s4u	1	-55.26	-52.35	-2.91	53.74
6s5k	1	-54.84	-52.50	-2.33	45.22
3l0e	1	-54.74	-50.06	-4.68	20.85
	2	-53.90	-49.22	-4.68	19.52
	3	-53.64	-48.96	-4.68	17.56
	4	-53.57	-48.89	-4.68	18.74
	5	-53.01	-48.33	-4.68	22.28
5jy3	1	52.50	54.05	-1.54	97.68
1p8d	1	-57.03	-54.06	-2.97	57.21
	2	-51.34	-50.30	-1.04	54.05
	3	-48.98	-45.79	-3.19	52.52
	4	-44.35	-42.38	-1.97	26.31
1upv	1	-59.90	-57.73	-2.17	32.92
	2	-39.69	-37.65	-2.04	60.10
1pq9	1	51.79	51.92	-0.13	76.81
1upw	1	-48.25	-41.86	-6.40	56.75
6k9h	1	-35.15	-40.54	5.40	50.34
1pqc-B	1	-25.96	-25.68	-0.27	22.47
4rak	1	-16.98	-15.89	-1.09	27.52
3kfc	1	-6.26	-6.03	-0.23	74.18
	2	-5.26	0.69	-5.95	31.07
6k9g	1	35.45	38.61	-3.16	57.48

vdW Van Der Waals force, ES Electrostatic.

Table S4. Grid Scores between human LXRα and CER(d18:1/18:1) (kcal/mol)

PDB ID	Pose	Grid Score	Grid vdW	Grid_es	Internal energy
**3ipu**	**1**	**-100.96**	**-95.17**	**-5.79**	**22.54**
3ips	1	-99.58	-97.12	-2.46	27.03
	2	-98.48	-95.46	-3.02	23.71
5hjs	1	-94.91	-91.15	-3.76	23.15
3ipq	1	-93.45	-87.3	-6.14	25.69
5avi	1	-90.54	-89.39	-1.15	42.92
	2	-85.15	-84.19	-0.96	44.83
5avl	1	-87.93	-85.95	-1.98	26.16
1uhl	1	-78.94	-78.07	-0.87	28.61
	2	-72.76	-71.23	-1.53	24.56

vdW Van Der Waals force, ES Electrostatic.

Table S5. The targeted sequences of gRNA.

Name	Number	Targeted sequence
Acer3 (mouse)	gRNA1	CAAGCAGGAAGGCTCATTGTGGG
	gRNA2	TCTCTGTACTGTAGTATGAATGG
	gRNA3	TCAAGCAGGAAGGCTCATTGTGG
	gRNA4	GAAACTACCCTCAAACTTTATGG

Table S6. The targeted sequences of AAVs.

Name	Number	Targeted sequence
Sult2a1 (mouse)	shCON	5′ - TTCTCCGAACGTGTCACT - 3′
	shSult2a1	5′ - GGAAGGACCACGACTCATAAC - 3′
Lxrβ (mouse)	shCON	5′ - CCTAAGGTTAAGTCGCCCTCG - 3′
	shLxrβ	5′ - TGAGATCATGTTGCTAGAAAC - 3′

Table S7. The targeted sequences of lentivirus (LV) and siRNA.

Name	Number	Targeted sequence
ACER3 (human)	LV-ACER3	5' - GATCAAGAACTCAGTAAACTA - 3'
SULT2A1 (human)	siRNA 1	5' - GCAUAGCUUUCCCUACUAUTT - 3'
	siRNA 2	5' - GGUCAUGGUUUGACCACAUTT - 3'
	siRNA 3	5' - CCGAAGAACUGAACUUAAUTT - 3'
LXRβ (human)	siRNA 1	5' - GGAAGAAGAAGAUUCGGAATT - 3'
	siRNA 2	5' - CCAACUGCAGUGCAACAAATT - 3'

	siRNA 3	5' - GCCAGAUGGACGCUUUCAUTT - 3'
LXRα (human)	siRNA	5' - CCUCAAGGAUUUCAGUUAUTT - 3'

Table S8. Primers for qPCR assays.

Gene (Mouse)	qPCR primer sequence
Actb	5' - GATGTATGAAGGCTTTGGTC - 3'<br>5' - TGTGCACTTTTATTGGTCTC - 3'
Acer3	5' - TGTGATTCACTGAGGAACTTTCG - 3'<br>5' - AGAAACTTCACTTTTGGCCTGTA - 3'
Sult2a1	5' - CCTCAAAGGAAATGTTCTATTCGGA - 3'<br>5' - TCCAGCTCATCTGGCCCTAA - 3'
Sult2a2	5' - TCCTCCAAGGAAATGGTACAACT - 3'<br>5' - TCCTGGAAAACTTTATCGAAGGCT - 3'
Sult2a3	5' - CATTTCCTCTCATCTTCCTGTCC - 3'<br>5' - GATCCTGGATTCTTCACAAGGTT - 3'
Sult2a4	5' - AACTGTGTCCATTCAGGACCG - 3'<br>5' - CGCCTTGGCCTTAGAACTGA - 3'
Sult2a5	5' - CCTCTCAGCATTTGTTATAAGTTGA - 3'<br>5' - GCAGGAAAAGGTATGCCTTCAAA - 3'
Cxcl2	5' - GAGCTTGAGTGTGACGCCCCCAGG - 3'<br>5' - TCGGATACTTCAGCGTCAGGA - 3'
Cxcr2	5' - ATGCCCTCTATTCTGCCAGAT - 3'<br>5' - GTGCTCCGGTTGTATAAGATGAC - 3'
Ly6G	5'- GACTTCCTGCAACACAACTACC - 3'<br>5' - ACAGCATTACCAGTGATCTCAGT - 3'
Mpo	5' - AGTTGTGCTGAGCTGTATGGA - 3'<br>5' - CGGCTGCTTGAAGTAAAACAGG - 3'
Tnf-α	5' - CCCTCACACTCAGATCATCTTCT - 3'<br>5' - GCTACGACGTGGGCTACAG - 3'
Il-6	5' - TAGTCCTTCCTACCCCAATTTCC - 3'<br>5' - TTGGTCCTTAGCCACTCCTTC - 3'
Collagen1	5' - TGGTCCCAAAGGTTCTCCTGGT - 3'<br>5' - TTAGGTCCAGGGAATCCCATCACA - 3'
Collagen3	5' - CTGTAACATGGAAACTGGGGAAA - 3'<br>5' - CCATAGCTGAACTGAAAACCACC - 3'
Lxrα	5' - CCTTCCTCAAGGACTTCAGTTACAA - 3'<br>5' - CATGGCTCTGGAGAACTCAAAGAT - 3'
Lxrβ	5' - GCTCAGGAGCTGATGATCCA - 3'<br>5' - GCGCTTGATCCTCGTGTAG - 3'
Fxr	5' - TTAGTCTTCACCACAGCCACC - 3'<br>5' - ACCTGTATACATACATTCAGCCAAC - 3'
Rxrα	5' - CTTTGACAGGGTGCTAACAGAGC - 3'<br>5' - ACGCTTCTAGTGACGCATACACC - 3'
Pxr	5' - AGAGATCATCCCTCTTCTGCCAC - 3'<br>5' - GATCTGGTCCTCAATAGGCAGGT - 3'

Car	5' - GGAGCGGCTGTGGAAATATTGCAT - 3'<br>5' - TCCATCTTGTAGCAAAGAGGCCCA - 3'
Vdr	5' - GATGCCCACCACAAGACCTA - 3'<br>5' - CGGTTCCATCATGTCCAGTG - 3'
Pparγ	5' - GCTTCCACTATGGAGTTCATGCTT - 3'<br>5' - ATCCGGCAGTTAAGATCACACCTA - 3'
Erα	5' - TCCAGCAGTAACGAGAAAGGA - 3'<br>5' - AGCCAGAGGCATAGTCATTGC - 3'
Erγ	5' - TCAAAGCCCTCACCACACTCT - 3'<br>5' - GCCAGGGACAGTGTGGAGAA - 3'
Cyp7a1	5' - CAAGAACCTGTACATGAGGGAC - 3'<br>5' - CACTTCTTCAGAGGCTGCTTTC - 3'
Cyp27a1	5' - GCCTCACCTATGGGATCTTCA - 3'<br>5' - TCAAAGCCTGACGCAGATG - 3'
Cyp8a1	5' - GCCCTTACTCCAAATCCTACCA - 3'<br>5' - TCGCACACATGGCTCGAT - 3'
Srd5b1	5' - TAACCAGGTGGAGTGCCACCCG - 3'<br>5' - CCATGATGGGTTGCGGCAGGT - 3'
Baat	5' - AGCACCACTCCTCACTTCCATAG - 3'<br>5' - TCCATCCTCCTGTATTTTCTTGTG - 3'
Abcc2	5' - CAAATCCAATTCTCTACCTATGCAC - 3'<br>5' - GCCTGCAGTGTTGGATCA - 3'
Asbt	5' - GGAACTGGCTCCAATATCCTG - 3'<br>5' - GTTCCCGAGTCAACCCACAT - 3'
Ntcp	5' - GGCCACAGACACTGCGCT - 3'<br>5' - AGTGAGCCTTGATCTTGCTGAACT - 3'
Oatp1b2	5' - CCCGTGACTAATCCAACAACA - 3'<br>5' - GCTTCTCAGAGACCATAGAAAACC - 3'
Ostβ	5' - GAGCATCCTGGCAAACAGA - 3'<br>5' - TGCAGGTCTTCTGGTGTTTCT - 3'
Bsep	5' - TGAATGGACTGTCGGTATCTGTG - 3'<br>5' - CCACTGCTCCCAACGAATG - 3'
Smpd1	5' - TGGGACTCCTTTGGATGGG - 3'<br>5' - CGGCGCTATGGCACTGAAT - 3'
Smpd2	5' - GCCCAGTTCATCCACCAC - 3'<br>5' - CCTCAGTCTCAACGAAAGC - 3'
Smpd3	5' - TCATGGACGTGGCCTATC - 3'<br>5' - ACCTGCACCTTGAGAAACAG - 3'
Smpd4	5' - GGAATCTCCGATGCCTACA - 3'<br>5' - ATCATTGGACCACTTGGGT - 3'
Smpd5	5' - ATGAGTCTCCCTGACATTTCGC - 3'<br>5' - GGACCAGTAAGTCGGGAAAAG - 3'
Asah1	5' - AATAACACTTGGGTTGTCAC - 3'<br>5' - TAGGATACCCAGATAACCAC - 3'
Asah2	5' - AGAGAGAGCAAGGTATTCTTC - 3'<br>5' - ACTATTCACAAAGTGGTTGC - 3'
Cerk	5' - ATCTCCACGGGACAATAAA - 3'<br>5' - GGCCATACAGGGCTTTC - 3'
Cers1	5' - CTCATTGCCTCTTCCTACGC - 3'

	5' - CAGCTGCACATCGCTGAC - 3'
Cers2	5' - TCATCCCTTCTCAGTATTGGT - 3'<br>5' - ATCCTTTCGCTTGACATCAG - 3'
Cers3	5' - CAGGCGAGGAGTATCCTGTG - 3'<br>5' - CTCTCCGACCAGAACCATTTTC - 3'
Cers4	5' - ACCCTGAATTTGTCCCTGTA - 3'<br>5' - CTTGAAGTCCTTGCGTTTG - 3'
Cers5	5' - CGGGGAAAGGTGTCTAAGGAT - 3'<br>5' - GTTCATGCAGTTGGCACCATT - 3'
Cers6	5' - TGTGCCATAGCCCTCAAC - 3'<br>5' - CTCCGAACATCCCAGTCC - 3'
Acer1	5' - ATGCTCATAGGTCTGTTCTC - 3'<br>5' - AGTGGTTATAGTTACCAGGC - 3'
Acer2	5' - GTGTGGCATATTCTCATCTG - 3'<br>5' - TAAGGGACACCAATAAAAGC - 3'
S1pr1	5' - ATGGTGTCCACTAGCATCCC - 3'<br>5' - CGATGTTCAACTTGCCTGTGTAG - 3'
S1pr2	5' - TTAACTCCCGTGCAGTGGTTT - 3'<br>5' - GCCAGGAGGCTAAAGACCG - 3'
S1pr3	5' - ACTCTCCGGGAACATTACGAT - 3'<br>5' - CCAAGACGATGAAGCTACAGG - 3'
S1pr4	5' - GTCAGGGACTCGTACCTTCCA - 3'<br>5' - GATGCAGCCATACACACGG - 3'
S1pr5	5' - GCTTTGGTTTGCGCGTGAG - 3'<br>5' - GGCGTCCTAAGCAGTTCCAG - 3'
Sgpp1	5' - GAGCAACTTGCCGCTCTACTA - 3'<br>5' - GGTCGAGATTCCAGATCCAGAA - 3'
Sgpp2	5’ - TTCACCCACTGGAATATCGACC - 3’<br>5’ - AAGTCTCACAACGGGAGGAAA - 3’
Sphk1	5' - ACAGACCATCCAAAGGTAGTTT - 3'<br>5' - CTCTATTCTGTGCTCAGTCTGTC - 3'
Sphk2	5'- GTACTCATGTTGGGCATCTT - 3'<br>5' - CATACTCCACTAACTCCCCA - 3'
Cert	5' - AGTGCCTCTGACGATGTTCAC - 3'<br>5' - ACCAGTTGCCAATTTGCATCA - 3'
Cerkl	5' - GAAGCATGGCTCTTAGGGT - 3'<br>5' - CTCCTCCTGTGGGCTGTAT - 3'
Degs1	5'- GAATGGGTCTACACGGACCAG - 3'<br>5'-AGTCATGGAGTGGTTAAGGCA - 3
Degs2	5' - TCTCGCACAATACTGCCTTTG - 3'<br>5' - TAGCGTAAGGTAGGCCAATGG - 3'
Spltc1	5' - TACGAGGCTCCAGCATACC - 3'<br>5' - TCAGAACGCTCCTGCAACT - 3'
Spltc3	5' - ACATCCATGAGTCCCGTAG - 3'<br>5' - TCCATACCTCCAATGTTCC - 3'
Samd8	5' - CAGACCTACCCACCACTCC - 3'<br>5' - TAGCACAGAATCACGCCAC - 3'
Ugcg	5' - GCTTCGTGCTCTTCGTGG - 3'<br>5' - TTGCCTTCTTGTTGAGGTGT - 3'

B4galt6	5' - ACGGAACAGATTATCCTGAAGGC - 3'<br>5' - GAAGTTTTGCGGAAGATACGTTG - 3'
Gba	5' - GCCAGGCTCATCGGATTCTTC - 3'<br>5' - CACGGGGTCAAGAGAGTCAC - 3'
Gba2	5' - CGTCCTTTGCCCTCGTC - 3'<br>5' - TGCCACCACTCCACTCATC - 3'
Sgms2	5' - TGGTATTGGTTGGGTTATGG - 3'<br>5' - CGGGCACAGGTAACGTAGTG - 3'
Enpp7	5' - CGGCAAATACATCGAGAACC - 3'<br>5' - CTCTGGATACCGAGCGTGGC - 3'
Galc	5' - CCGATTTCCTCTTCCTTGCT - 3'<br>5' - GGTTCAATATGCGACTCCAA - 3'
Sgpl1	5' - GAACCGACCTCCTCAAGCT - 3'<br>5' - TCATACACCCAGACTATCAGC - 3'
Gla	5' - ACCCTTTCATAAGCCCAATT - 3'<br>5' - GGTCCAGCGACTTCAACAA - 3'
Scd1	5' - TTCTTGCGATACACTCTGGTGC - 3'<br>5' - CGGGATTGAATGTTCTTGTCGT- 3'
Fasn	5' - GGAGGTGGTGATAGCCGGTAT- 3<br>5' - TGGGTAATCCATAGAGCCCAG- 3
Srebp1c	5' - TGACCCGGCTATTCCGTGA - 3<br>5' - CTGGGCTGAGCAATACAGTTC - 3
Srebp2	5' - GCAGCAACGGGACCATTCT - 3'<br>5' - CCCCATGACTAAGTCCTTCAACT - 3'
Chrebp	5' - CAAGTTGCTATGCCGGGACAA - 3'<br>5' - CCTCCGTTGCACATACTGAATG - 3'
Abca1	5' - GCTTGTTGGCCTCAGTTAAGG - 3'<br>5' - GTAGCTCAGGCGTACAGAGAT - 3'
Srebp1	5' - GCAGCCACCATCTAGCCTG - 3'<br>5' - CAGCAGTGAGTCTGCCTTGAT - 3'
Pparα	5' - AACATCGAGTGTCGAATATGTGG - 3'<br>5' - CCGAATAGTTCGCCGAAAGAA - 3'
**Gene (Human)**	**qPCR primer sequence**
ACTB	5' - CATGTACGTTGCTATCCAGGC - 3'<br>5' - CTCCTTAATGTCACGCACGAT - 3'
ACER3	5' - ACTACTCCGTGACCTGGTACA - 3'<br>5' - GCACCGAACATTGGAGGTATAAT - 3'
SULT2A1	5' - CTGGGAAAGACGTTAGAACCC - 3'<br>5' - AAGTTGTGCTTTGTCCACTACAT - 3'
LXRβ	5' - CTCCTGAAGGCATCCACTATCG - 3'<br>5' - GGTGGAAGTCGTCCTTGCTGTA - 3'
LXRα	5' - CCTTCAGAACCCACAGAGATCC - 3'<br>5' - ACGCTGCATAGCTCGTTCC - 3'
SPTLC1	5' - GGTGGAGATGGTACAGGCG -3'<br>5’ - TGGTTGCCACTCTTCAATCAG -3’
SPTLC2	5' - TGGGTTCCTACAACTATCTTGGA -3'<br>5’ - CATACGCCATAGCAGCTTCTAC -3’
SPTLC3	5' - GGAATTGGAACCCTGTTTGGC -3'

	5’ - GTCTCTGATTCGCATGTAAAGGT -3’
DEGS1	5' - CTTTTATGCCTTTCGACCTCTGT -3'<br>5’ - CTGTGCCACGGTATTGATAACT -3’
DEGS2	5' - GCGGGTGTACAGGCTGGCAAAAGA -3'<br>5’ - ACAAGGGCAGCAGTCCAGAGCACA -3’
CERS1	5' - TACAGTGCCTACCTGCTGTTT -3'<br>5’ - TAGCGTAGCGTAGATGGAGTG -3’
CERS2	5' - GGTAGAGCGTTGGTTCCGTC -3'<br>5’ - GGCAATGAAGGCAATCAGGTAA -3’
CERS3	5' - AACATTCCACAAGGCAACCAT -3'<br>5’ - GACTCCTAAACCATCTTTCCACC -3’
CERS4	5' - TCGGTCCTGTACCACGAGTC -3'<br>5’ - GCCTGATTAGCAGTGAGAGGTAG -3’
CERS5	5' - GCTGCTCTTCGAGCGATTTAT -3'<br>5’ - CCTCCGATGGCGAAACCAG -3’
CERS6	5' - GCAGGGATCTTAGCCTGGTTC -3'<br>5’ - AAAAGCGAGATAGAGGTCCTCA -3’
CERK	5' - TATCAACCCGTTTGGAGGAAAAG -3'<br>5’ - ATGGAGGCTAAGGTGAACAGT -3’
CERKL	5' - TGGTGGAAGAACTTTGGCTCT -3'<br>5’ - AGGAATTGCCATAATGCTGACA -3’
CERT	5' - TCCATCTGTCTTAGCAAGGCT -3'<br>5’ - GCTGTTCAATGGCATCTATCCA -3’
ACER1	5' - GCTCCCGCTACATTTACGTTG -3'<br>5’ - GCTGAGCGTCATGTGGAAATAC -3’
ACER2	5' - TCCATGCAACCCTTAGTTTCTTG -3'<br>5’ - CTACCCCGGTCATTCCGAAAG -3’
ASAH1	5' - ATTGGCCCCAGCCTACTTTAT -3'<br>5’ - CCCTGCTTAGCATCGAGTTCAT -3’
ASAH2	5' - CCACCCGGTCAGCATGAAC -3'<br>5’ - GTGGTCCAAGAATGTTGGGG -3’
SPHK1	5' - AGAGTGGGTTCCAAGACACCT -3'<br>5’ - GGGTGCAGCAAACATCTCAC -3’
SPHK2	5' - ATGGCATCGTCACGGTCTC -3'<br>5’ - CTCCCAGTCAGGGCGATCTA -3’
SGPP1	5' - CCATTTGTGGACCTGATTGACA -3'<br>5’ - ACTTCCTAGTATCTCGGCTGTG -3’
SGPP2	5' - CAAGCCCGCTGAATCTCTCC -3'<br>5’ - GAGGATCAACACAATTCCCACT -3’
SGPL1	5' - GAAGATGCCCATTATTGGTCGT -3'<br>5’ - CGTCCATAGAGCTGTACTCCTT -3’
GALC	5' - CGAACTCTTCAAGGTGGTTGAT -3'<br>5’ - GCCTGCACCCATGTCACTATT -3’
GLA	5' - CTGAGGAACCCAGAACTACATCT -3'<br>5’ - GGTAGGCGTCCTTGCCAAT -3’
GBA	5' - CATCCGCACCTACACCTATGC -3'<br>5’ - TGAGCTTGGTATCTTCCTCTGG -3’
GBA2	5' - CAAGCTAACAACGTCTCCCTAAG -3'<br>5’ - GATCATGTCGATGAAAGGTGTCT -3’

B4GALT6	5' - AACGGTACAGATTATCCCGAAGG -3'<br>5’ - AGGAATCCTCGCATATAAGGCA -3’
SMPD1	5' - ATCTGCTGAAGATAGCACCACC -3'<br>5’ - CTTCGGCACAGTAGGCAAAG -3’
SMPD2	5' - TCAATGGCTACCCCTACATGA -3'<br>5’ - ATGCCACTTAGATGGAGCACC -3’
SMPD3	5' - GCTGCCCTTTGCGTTTCTC -3'<br>5’ - TCCAGCCGTGAATAGATGTAGG -3’
SMPD4	5' - CCACGTCCGTACTTCAGACTG -3'<br>5’ - TCGCTTTAGGAGGCTAGTGTG -3’
ENPP7	5' - AAAAATGAGACGGAGTGGAGAGC -3'<br>5’ - CCGAAGTAGAGTGTGACCAGA -3’
UGCG	5' - GAATGGCCGTCTTCGGGTT -3'<br>5’ - AGGTGTAATCGGGTGTAGATGAT -3’
NAAA	5' - TGACAGTGGATGTGCAATTCTT -3'<br>5’ - GCCTTTATCTCGTTCATCACCAG -3’
SAMD8	5' - ATGGCAGGTCCTAATCAACTCT -3'<br>5’ - AGACCGGAGATCATATTCAGTCA -3’
SGMS1	5' - CAGCATCAAGATTAAACCCAACG -3'<br>5’ - TGGTGAGAACGAAACAGGAAAG -3’
SGMS2	5' - CAAATTGCTATGCCCACTGAATC -3'<br>5’ - GTTGTCAAGACGAGGTTGAAAAC -3’

Table S9. Key resource.

**Reagent or resource**	**Source**	**Identifier**
Antibodies		
Anti-ACER3 Rabbit mAb	Sigma-Aldrich	Cat: #HPA070087
Anti-αSMA Rabbit mAb	Cell Signaling Technology	Cat: #19245S
Anti-SULT2A1 Rabbit mAb	Abcam	Cat: #ab194113
Anti-LXRβ Rabbit pAb	Abcam	Cat: #ab28479
Anti-LXRα Rabbit mAb	Abcam	Cat: #ab176323
Anti-FXR Mouse mAb	Cell Signaling Technology	Cat: #72105S
Anti-PXR Rabbit pAb	Abcam	Cat: #ab192579
Anti-CAR Rabbit pAb	Abcam	Cat: #ab186869
Anti-RXRα Rabbit mAb	Abcam	Cat: #ab125001
Anti-LY6G Rabbit mAb	Abcam	Cat: #ab238132
Anti-ALB Mouse mAb	Proteintech	Cat: #16475-1-AP
Anti-PCNA Rabbit mAb	Cell Signaling Technology	Cat: #13110S
Anti-B4GALT6 Rabbit mAb	Proteintech	Cat: # 20148-1-AP
Anti-SREBP1 Rabbit mAb	Abcam	Cat: #ab313881
Anti-PPARα Rabbit mAb	Abcam	Cat: #ab314112
Anti-DEGS2 Rabbit pAb	Thermo Fisher Scientific	Cat: #PA5-24082
Anti-SMPD3 Mouse pAb	Thermo Fisher Scientific	Cat: #PA5-117447
Anti-GLA Mouse mAb	Proteintech	Cat: #66121-1-IG

Anti-CERS3 Rabbit mAb	Thermo Fisher Scientific	Cat: #PA5-113105
Anti-Cleaved-caspase 3 Rabbit mAb	Cell Signaling Technology	Cat: #9664S
Anti-Cleaved-PARP Rabbit mAb	Cell Signaling Technology	Cat: #5625S
Anti-Histone H3 Rabbit pAb	Abcam	Cat: #ab1791
Anti-FLAG Rabbit mAb	Abcam	Cat: #ab205606
Anti-GAPDH Mouse mAb	Abcam	Cat: #ab8245
Anti-β-Tubulin Rabbit mAb	Abcam	Cat: #ab68193
Anti-β-Actin Rabbit mAb	Cell Signaling Technology	Cat: #4970S
Anti-rabbit IgG HRP	Cell Signaling Technology	Cat: #7074
Anti-mouse IgG HRP	Cell Signaling Technology	Cat: #7076
Alexa Fluor® 488-conjugated Rabbit antibody	Abcam	Cat: #ab150077
Alexa Fluor® 594-conjugated Mouse antibody	Abcam	Cat: #ab150116
Bacterial and virus strains		
Plasmid-pcDNA$^{TM3.1}$-Flag-LXRβ	This paper	N/A
Plasmid-pGL3-SULT2A1-Luciferase	This paper	N/A
Plasmid-pRL-TK	This paper	N/A
Lentivirus shACER3	This paper	See Table S7
SULT2A1 siRNA	This paper	See Table S7
LXRβ siRNA	This paper	See Table S7
LXRα siRNA	This paper	See Table S7
Sult2a1-AAV	This paper	See Table S6
Lxrβ-AAV	This paper	See Table S6
Biological samples		
Human-derived Clinical Sample	Southern Medical University affiliated Nanfang Hospital	N/A
Chemicals, peptides, and recombinant proteins		
Carboxymethylcellulose	Sigma-Aldrich	Cat: #419273
4% paraformaldehyde	Sigma-Aldrich	Cat: #P6148
Tissue-Tek OCT compound	Sakura Finetek	Cat: #4583
Dulbecco's modified Eagle's medium	Gibco	Cat: #C11995500BT
Roswell Park Memorial Institute medium 1640	Gibco	Cat: #C11875500BT
Fetal bovine serum	Gibco	Cat: #10099141
Penicillin/Streptomycin	Gibco	Cat: #15140122
Lithocholic acid	Sigma-Aldrich	Cat: #L6250
DMSO	Sigma-Aldrich	Cat: #D2650
Puromycin	Gibco	Cat: #A1113803

Minimal essential medium	Gibco	Cat: #11095080
Dodecane	Sigma-Aldrich	Cat: #297879
Oil Red O	Sigma-Aldrich	Cat: #O1391
DAPI	Abcam	Cat: #ab285390
TriZol	Invitrogen	Cat: #15596026
Glycochenodeoxycholic acid-d4	Avanti Polar Lipids	Cat: #330273
Glycocholic acid-d4	Avanti Polar Lipids	Cat: #330277
Glycodeoxycholic acid-d4	Avanti Polar Lipids	Cat: #330273
Cholic acid-d4	Avanti Polar Lipids	Cat: #330256
Ursodeoxycholic acid-d4	Avanti Polar Lipids	Cat: #330260
Chenodeoxycholic acid-d4	Avanti Polar Lipids	Cat: #330259
Deoxycholic acid-d4	Avanti Polar Lipids	Cat: #330257
Lithocholic acid-d4	Avanti Polar Lipids	Cat: #330258
Acetonitrile	Sigma-Aldrich	Cat: #34851
Lithocholic acid 3-sulfate	Sigma-Aldrich	Cat: #700317
Taurolithocholic acid 3-sulfate	Sigma-Aldrich	Cat: #T0512
Cholic acid 3-sulfate	Sigma-Aldrich	Cat: #700323
Taurocholic acid 3-sulfate	BePure	Cat: #MU-1055
Glycodeoxycholic acid 3-sulfate	BePure	Cat: # MU-1050
Taurochenodeoxycholic acid 3-sulfate	BePure	Cat: # MU-1042
Methyl tert-butyl ether	Sigma-Aldrich	Cat: #650560
CER(d18:1/17:0)	Avanti Polar Lipids	Cat: #860517
SPH(d17:1)	Avanti Polar Lipids	Cat: #860640
S1P(d17:1)	Avanti Polar Lipids	Cat: #860641
CER(d18:1/6:0)	Avanti Polar Lipids	Cat: #860506
CER(d18:1/16:0)	Avanti Polar Lipids	Cat: #860516
CER(d18:1/18:0)	Avanti Polar Lipids	Cat: #860518
CER(d18:1/18:1)	Avanti Polar Lipids	Cat: #860519
CER(d18:1/20:0)	Avanti Polar Lipids	Cat: #860520
CER(d18:1/22:0)	Avanti Polar Lipids	Cat: #860501
CER(d18:1/24:0)	Avanti Polar Lipids	Cat: #860524
CER(d18:1/24:1)	Avanti Polar Lipids	Cat: #860525
SPH(d18:1)	Avanti Polar Lipids	Cat: #860490
S1P(d18:1)	Avanti Polar Lipids	Cat: #860492
d7-24-hydroxcholesterol	Avanti Polar Lipids	Cat: #700018
d7-7β-hydroxycholesterol	Avanti Polar Lipids	Cat: #700044
d6-25-hydroxycholesterol	Avanti Polar Lipids	Cat: #700053
d6-27-hydroxycholesterol	Avanti Polar Lipids	Cat: #700059
d7-7-keto-cholesterol	Avanti Polar Lipids	Cat: #700046
d7-7α-hydroxy-cholestenone	Avanti Polar Lipids	Cat: #700112
d6-TMAS	Avanti Polar Lipids	Cat: #700074

d7-4β-hydroxycholesterol	Avanti Polar Lipids	Cat: #700042
d6-24,25-epoxycholesterol	Avanti Polar Lipids	Cat: #700048
d6-desmosterol	Avanti Polar Lipids	Cat: #700040
d3-3β-7α-dihydroxycholestrol-5-enoic acid	Avanti Polar Lipids	Cat: #700224
Dimethylformamide	Sigma-Aldrich	Cat: #270547
Recombinant human LXRβ	AntibodySystem	Cat: #YHF08201
Critical commercial assays		
RNA iMAX transfection reagent	Invitrogen	Cat: #13778150
Lipofectamine 3000	Invitrogen	Cat: #L3000015
Dual-luciferase assay kit	Promega	Cat: #E1960
RNA scope ISH kit	Advanced Cell Diagnostics	Cat: #322350
ALT Colorimetric Activity Assay Kits	Sigma-Aldrich	Cat: #E1960
AST Colorimetric Activity Assay Kits	Sigma-Aldrich	Cat: #MAK467
VECTASTAIN® Elite® ABC Kit	VECTOR	Cat: #PK-6100
DAB Peroxidase Substrate Kit	VECTOR	Cat: #PK-6100
Detergent-free MinuteTM Total Protein Extraction Kit	Invent Biotechnologies	Cat: #SN-006
EZview Red ANTI-FLAG M2 Affinity Gel	Sigma-Aldrich	Cat: #F2426
FLAG peptide	Sigma-Aldrich	Cat: #F4799
5 × PrimeScript RT Master Mix	TaKaRa	Cat: #RR036
RIPA buffer	Thermo Scientific	Cat: #89901
Nuclear and Cytoplasmic Protein Extraction Kit	Invent Biotechnologies	Cat: #NT-032
BCA protein determination kit	Thermo Fisher Scientific	Cat: #23227
PVDF membranes	Roche	Cat: #03010040001
ECL Prime Western Blotting Detection Reagent	Cytiva	Cat: #RPN2232
Experimental models: cell lines		
HepG2	Cell Bank of Chinese Academy of Science	TCHu72
Huh-7	Cell Bank of Chinese Academy of Science	TCHu182
Hep3B	Cell Bank of Chinese Academy of Science	TCHu106
MHCC97-H	Cell Bank of Chinese Academy of Science	SCSP-5092
Experimental models: organisms/strains		
C57BL/6J Mice	Southern Medical University	N/A
Global *Acer3* Deficient Mice	Southern Medical University	N/A
Hepatocyte-specific *Acer3*	Southern Medical University	See Tabel S5

Deficient Mice		
Oligonucleotides		
Primer sequences for cloning or sequencing	This paper	See Table S8
Software and algorithms		
Gen5	Biotek	https://www.biotek.com/products/software-robotics/
Image Pro Plus	Media Cybernetics	https://mediacy.com/image-pro/
StringTie	Johns Hopkins University Center for Computational Biology	https://ccb.jhu.edu/software/stringtie/
Lipid Search	Thermo Fisher Scientific	https://www.thermofisher.com/
UCSF Chimera	University of California, San Francisco	https://www.cgl.ucsf.edu
Discovery Studio	DiscoveryStudio Software	http://www.discoverystudio.net/
SWISS-MODEL	Biozentrum of the University of Basel	http://swissmodel.expasy.org/
BIA evaluation	Cytiva	https://www.cytivalifesciences.com.cn/
GraphPad Prism	GraphPad Software	https://www.graphpad.com/
Statistical Product and Service Solutions	IBM	https://www.ibm.com/cn-zh/spss

Table S10. Multiple reaction monitoring parameters of ceramides

Compound	t_R (min)	Precursor (m/z)	Product (m/z)	Collision Energy (V)
CER(d18:1/16:0)	6.97	538.7	264.3	25
CER(d18:1/18:0)	7.30	566.7	264.4	26
CER(d18:1/20:0)	7.60	594.7	264.3	27
CER(d18:1/22:0)	7.91	622.7	264.3	28
CER(d18:1/24:0)	8.20	650.7	264.3	29
CER(d18:1/18:1)	7.00	564.7	264.3	26
CER(d18:1/20:1)	7.31	592.7	264.3	27
CER(d18:1/22:1)	7.60	620.7	264.3	28
CER(d18:1/24:1)	7.90	648.6	264.3	29
CER(d18:1/26:1)	8.18	676.7	264.3	28
SPH(d18:1)	5.17	300.3	252.3	18
S1P(d18:1)	5.96	380.3	264.3	16

Table S11. Multiple reaction monitoring parameters of BA-sulfates

Compound	t_R (min)	Precursor (m/z)	Product (m/z)	Collision Energy (V)
T-LCA-S	4.34	280.8	97	44
T-CDCA-S	3.96	288.6	96.8	44
T-CA-S	3.73	297.1	97	47
LCA-S	5.49	455.3	97	41
CA-S	4.34	487.3	97	45
G-DCA-S	4.11	528.2	452.3	42

Supplementary Figures and Figure Legends

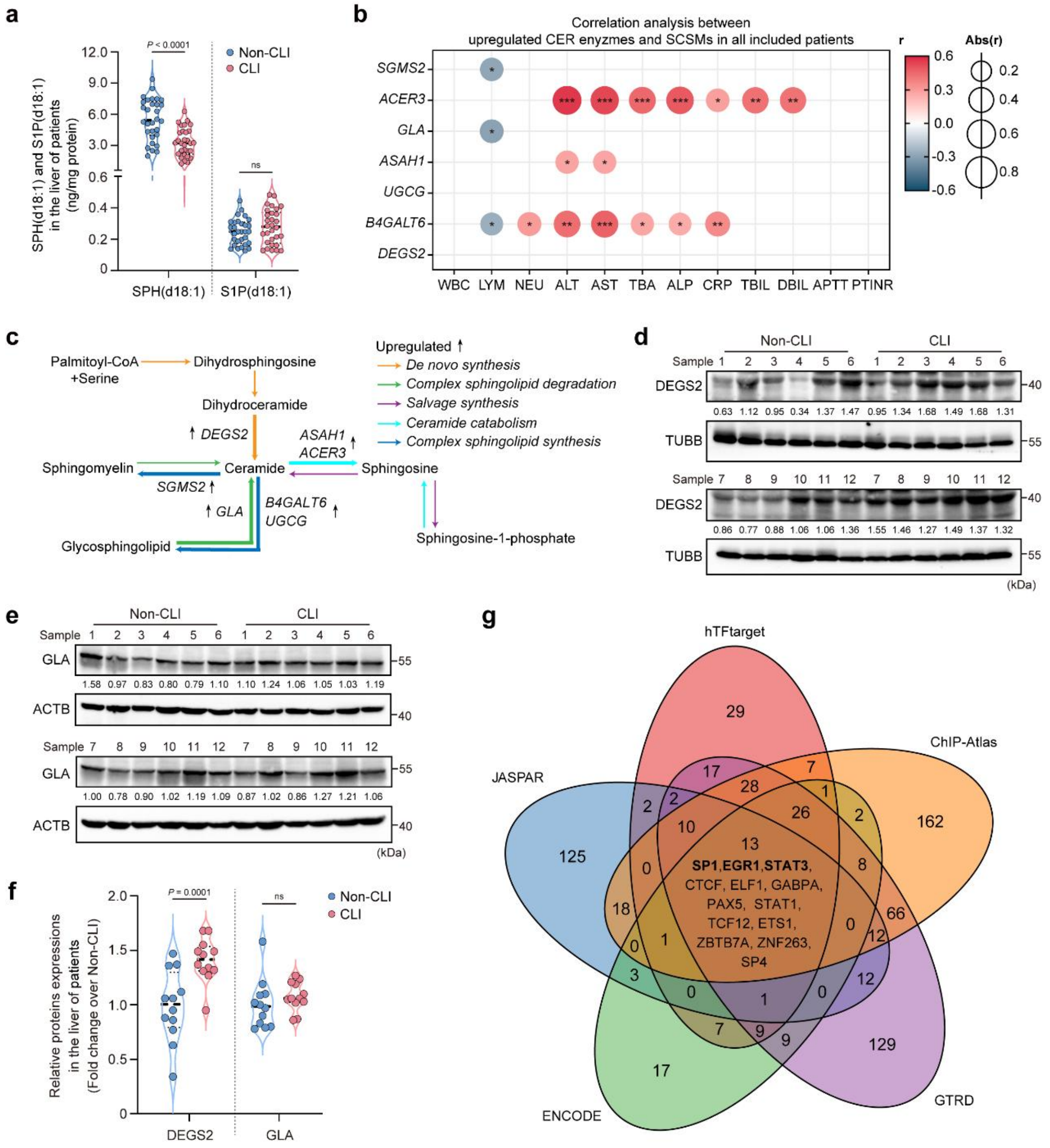

Figure S1. Sphingolipid metabolism in the liver of patients with or without CLI.

(**a**) Sphingosine (SPH) (d18:1) and sphingosine-1-phosphate (S1P) (d18:1) in the liver tissues of patients with non-cholestatic liver injury (CLI) and CLI (n = 30).

(**b**) Correlation between upregulated CER enzymes and serum cholestatic liver injury severity markers (SCSMs) in all patients.

(**c-f**) Sphingolipid metabolism in the liver tissues of patients. Schemed diagram of dysregulation in sphingolipid metabolic enzymes (**c**). Immunoblot of sphingolipid delta(4)-desaturase/C4-monooxygenase (DEGS2) (**d**) and galactosidase A (GLA) (**e**), and quantification of DEGS2 and GLA (**f**) in the liver tissues of patients (n = 12).

(**g**) Venn chart of alkaline ceramidase 3 (ACER3)-related potential transcription factor prediction from hTFtarget, ChIP-Atlas, GTRD, ENCODE, and JASPAR databases. Data are expressed as mean ± SD. Statistical significances were tested by the unpaired two-sided Student's *t*-test (**a**, **f**) and Spearman correlation test (**b**). $*P < 0.05$, $**P < 0.01$, $***P < 0.001$. Source data are provided as a Source Data file.

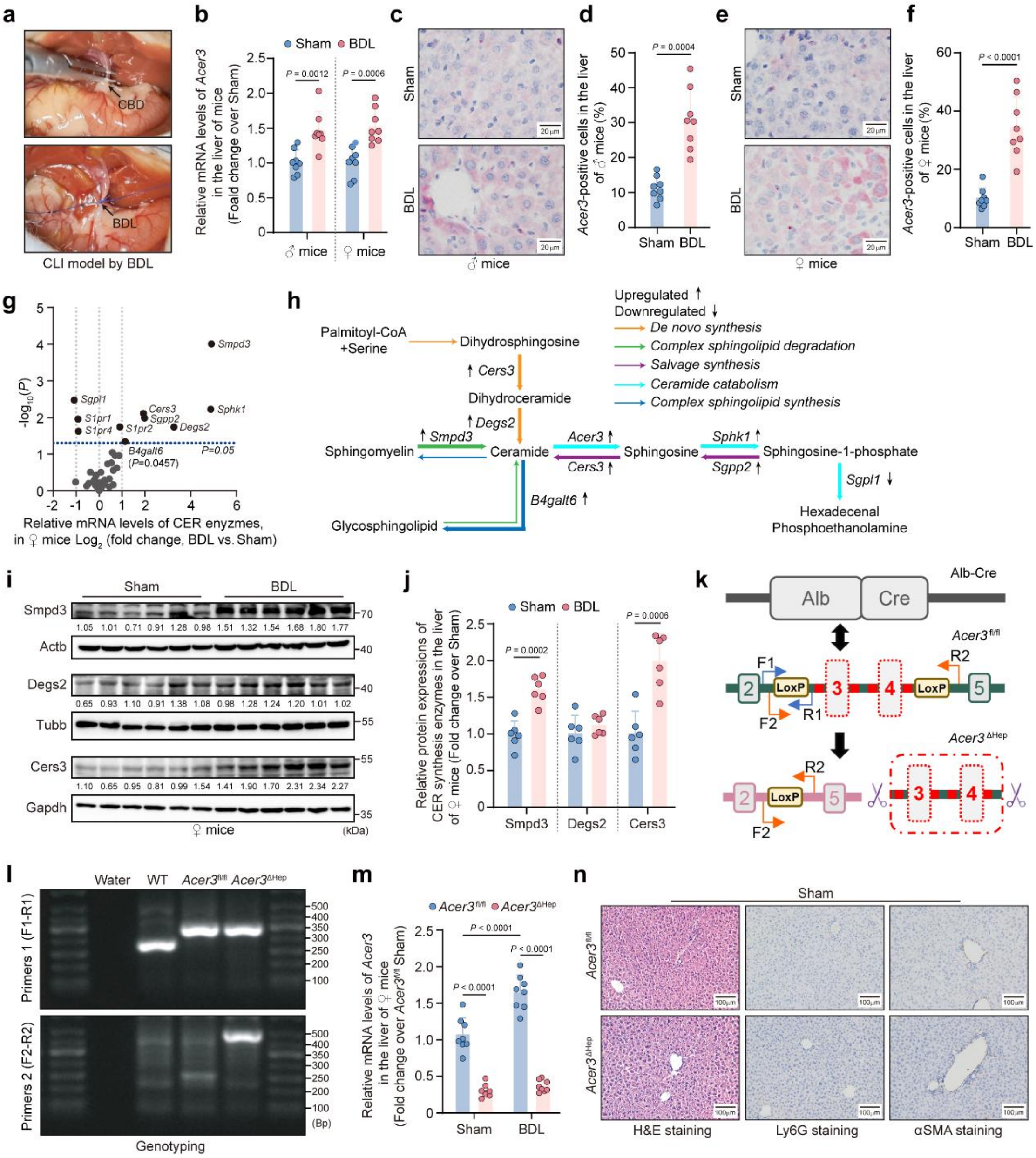

Figure S2. Dysregulation of sphingolipid metabolism in the liver of female mice after BDL and establishment of *Acer3*$^{fl/fl}$ and *Acer3*$^{\Delta Hep}$ mice.

(**a**) The mouse model of CLI was established by bile duct ligation (BDL).

(**b-f**) Hepatic *Acer3* expression in C57BL/6J wildtype (WT) mice (n = 8). *Acer3* mRNA levels (**b**). *in situ* hybridization (ISH) and quantification of *Acer3*-positive cells in the liver of male (**c** and **d**) and female (**e** and **f**) mice.

(**g**) Volcano plot of the mRNA levels of sphingolipid metabolic enzymes in the liver of female mice subjected to BDL and sham operation (n = 3).

(**h**) Schemed diagram of dysregulation in sphingolipid metabolism in the liver of female mice after BDL.

(**i** and **j**) Immunoblot of sphingomyelin phosphodiesterase 3 (Smpd3), Degs2, and ceramide synthase 3 (Cers3) (**i**) in the liver of female mice after BDL (n = 6). Quantification of Smpd3, Degs2, and Cers3 proteins (**j**).

(**k**) Schematic diagram depicting the design of *Acer3*$^{fl/fl}$ and *Acer3*$^{\Delta Hep}$ mice. Created in BioRender. Liao, L. (2025), https://BioRender.com/k27c243.

(**l**) Genotyping of mouse tail in *Acer3*$^{fl/fl}$ and *Acer3*$^{\Delta Hep}$ mice.

(**m**) The mRNA levels of *Acer3* in the liver of *Acer3*$^{fl/fl}$ and *Acer3*$^{\Delta Hep}$ female mice subjected to BDL or sham operation (n = 8).

(**n**) Hematoxylin and eosin (H&E) (left panel), lymphocyte antigen 6 complex locus G6D (Ly6G) (middle panel), and alpha-smooth muscle actin (αSMA) (right panel) staining in the liver sections of *Acer3*$^{fl/fl}$ and *Acer3*$^{\Delta Hep}$ female mice under basal conditions (n = 8).

Data are expressed as mean ± SD. Statistical significances were tested by the unpaired two-sided Student's *t*-test (**b**, **d**, **f**, **g**, **j**) and one-way ANOVA with Tukey's multiple comparisons (**m**). Source data are provided as a Source Data file.

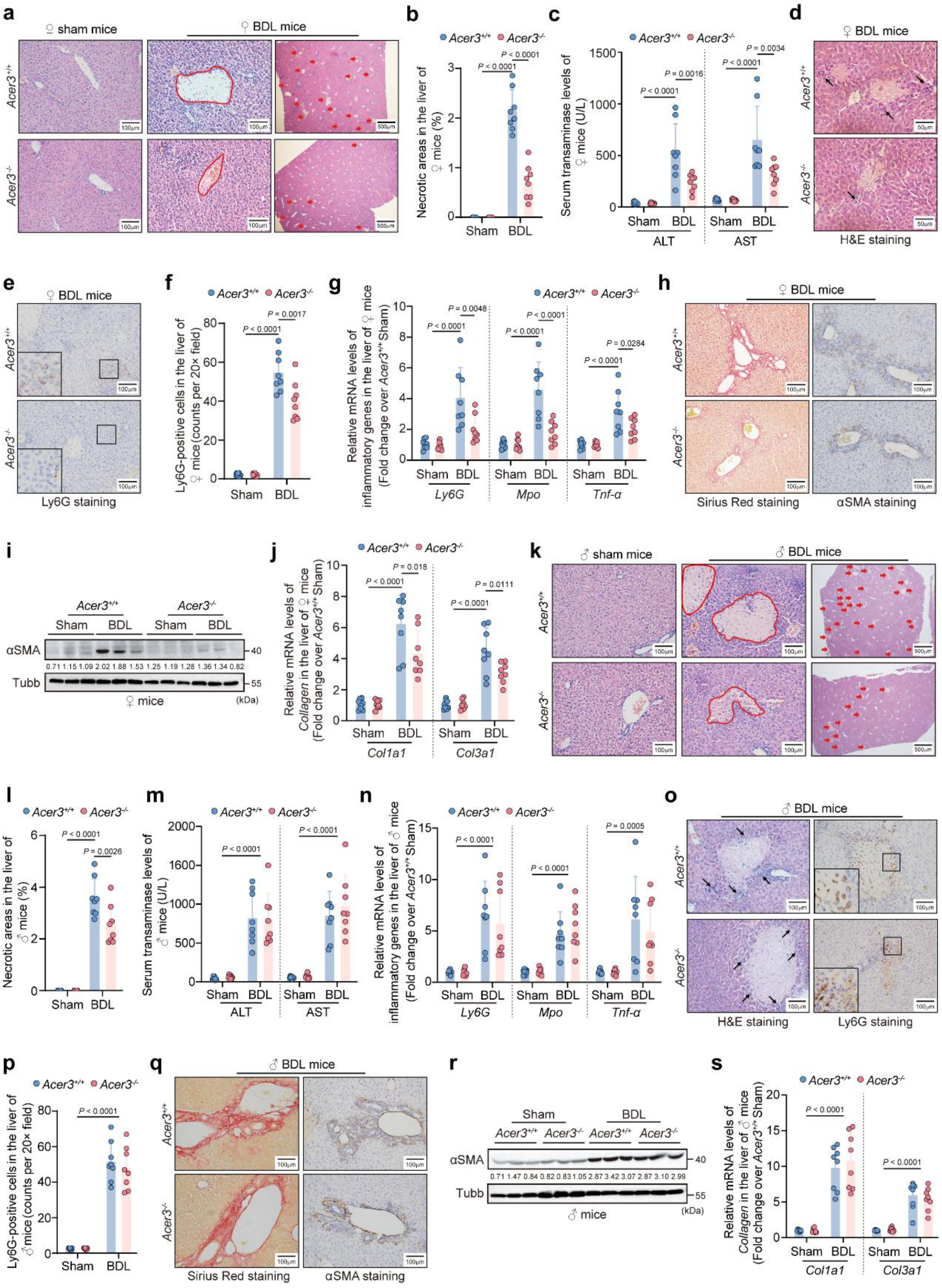

Figure S3. Global *Acer3* ablation attenuates CLI in female mice but does not substantially affect CLI in male mice.

(**a**-**j**) Examination of BDL-induced liver injury in *Acer3*$^{+/+}$ and *Acer3*$^{-/-}$ female mice (n = 8). H&E staining with the circle areas and red arrows indicating necrotic foci (**a**) and quantification of necrotic areas (**b**) in liver sections. Serum transaminase levels

(**c**). H&E-stained necrotic foci with black arrows indicating inflammatory cell infiltration in liver sections (**d**). Ly6G staining (**e**) and quantification of Ly6G-positive cells in liver sections (**f**). The mRNA levels of inflammatory genes in the liver (**g**). Sirius Red staining (left panel) and αSMA staining (right panel) in liver sections (**h**). αSMA immunoblot of the liver (**i**). The mRNA levels of *Collagen* in the liver (**j**).

(**k-s**) Examination of BDL-induced liver injury in $Acer3^{+/+}$ and $Acer3^{-/-}$ male mice (n = 8). H&E staining with the circle areas and red arrows indicating necrotic foci (**k**) and quantification of necrotic areas (**l**) in liver sections. Serum transaminase levels (**m**). The mRNA levels of inflammatory genes in the liver (**n**). H&E-stained necrotic foci with black arrows indicating inflammatory cell infiltration in liver sections (**o**, left panel). Ly6G staining (**o**, right panel) and quantification of Ly6G-positive cells in liver sections (**p**). Sirius Red staining (left panel) and αSMA staining (right panel) in liver sections (**q**). αSMA immunoblot of the liver (**r**). The mRNA levels of *Collagen* in the liver (**s**).

Data are expressed as mean ± SD. Statistical significances were tested by the one-way ANOVA with Tukey's multiple comparisons test (**b**, **c**, **f**, **g**, **j**, **l-n**, **p**, **s**). Source data are provided as a Source Data file.

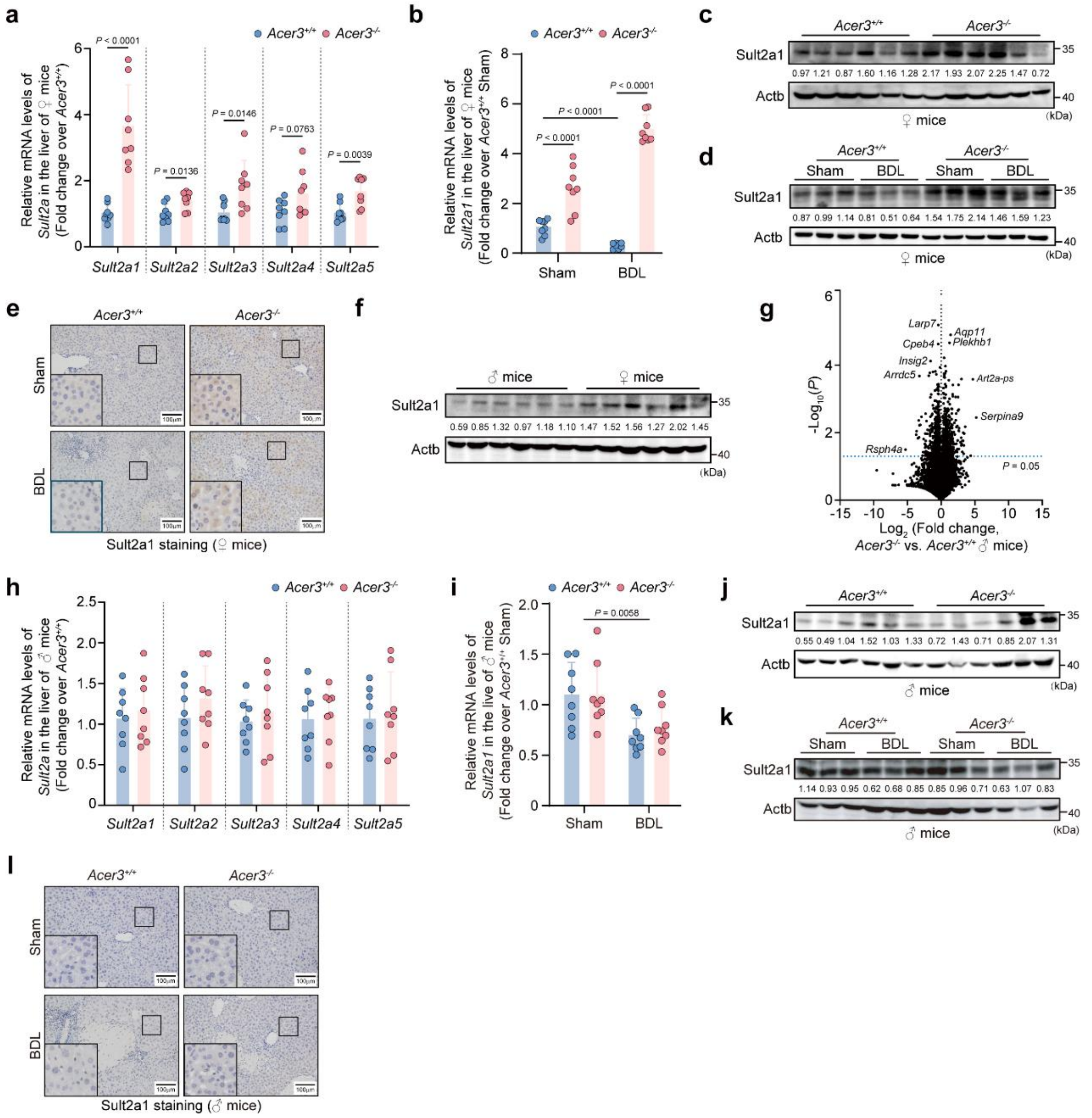

Figure S4. *Sult2a1* expression in the liver of female and male mice.

(**a**) The mRNA levels of sulfotransferase 2a (*Sult2a*) families in the liver of *Acer3*$^{+/+}$ and *Acer3*$^{-/-}$ female mice under normal conditions (n = 8).

(**b**-**e**) *Sult2a1* expression in the liver of *Acer3*$^{+/+}$ and *Acer3*$^{-/-}$ female mice subjected to sham operation or BDL (n = 8). mRNA levels of *Sult2a1* in the liver (**b**). Protein levels of *Sult2a1* in the liver under basal conditions (**c**) or after sham operation or BDL (**d**). Sult2a1 staining in the liver sections (**e**).

(**f**) Immunoblot of Sult2a1 in the liver of male and female C57BL6/J WT mice (n = 8).

(**g**) Volcano plot of differentially expressed genes (DEGs) in the liver of *Acer3*$^{+/+}$ and *Acer3*$^{-/-}$ male mice under basal conditions (n = 4).

(**h**) The mRNA levels of *Sult2a* families in the liver of male mice under basal

conditions (n = 8).

(**i-l**) *Sult2a1* expression in the liver of *Acer3*$^{+/+}$ and *Acer3*$^{-/-}$ male mice subjected to sham operation or BDL (n = 8). mRNA levels of *Sult2a1* in the liver (**i**). Protein levels of *Sult2a1* in the liver under basal conditions (**j**) or after sham operation or BDL (**k**). Sult2a1 staining in the liver sections (**l**).

Data are expressed as mean ± SD. Statistical significances were tested by the unpaired two-sided Student's t-test (**a**, **h**) and one-way ANOVA with Tukey's multiple comparisons test (**b**, **i**). Source data are provided as a Source Data file.

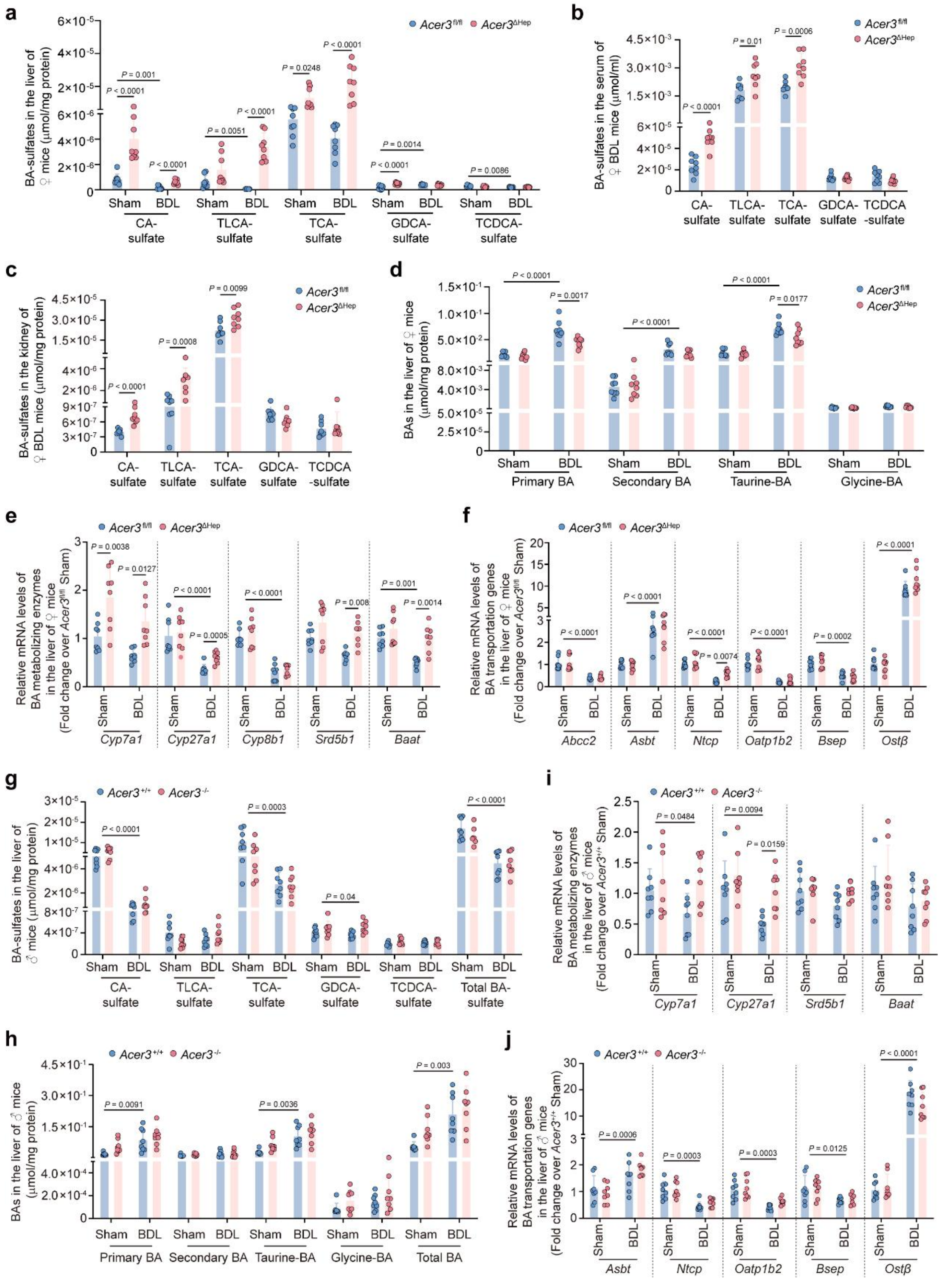

Figure S5. ***Acer3*** **ablation promotes Sult2a1-catalyzed BA sulfation and stabilizes BA metabolism in the cholestatic liver of female mice, but not in males.**

(**a**-**c**) Individual bile acid (BA)-sulfate species in the liver (**a**), serum (**b**), and kidney (**c**) of *Acer3*$^{fl/fl}$ and *Acer3*$^{\Delta Hep}$ female mice subjected to BDL or sham operation (n = 8). (**d**) Individual BA species in the liver of *Acer3*$^{fl/fl}$ and *Acer3*$^{\Delta Hep}$ female mice subjected

to BDL or sham operation (n = 8).

(**e** and **f**) The mRNA levels of genes involved in BA metabolism (**e**) and BA transportation (**f**) in the liver of *Acer3*$^{fl/fl}$ and *Acer3*$^{\Delta Hep}$ female mice subjected to BDL or sham operation (n = 8).

(**g** and **h**) Individual BA-sulfate species (**g**) and BA species (**h**) in the liver of *Acer3*$^{+/+}$ and *Acer3*$^{-/-}$ male mice subjected to BDL or sham operation (n = 8).

(**i** and **j**) The mRNA levels of genes involved in BA metabolism (**i**) and BA transportation (**j**) in the liver of *Acer3*$^{+/+}$ and *Acer3*$^{-/-}$ male mice subjected to BDL or sham operation (n = 8).

Data are expressed as mean ± SD. Statistical significances were tested by the one-way ANOVA with Tukey's multiple comparisons (**a-j**). Source data are provided as a Source Data file.

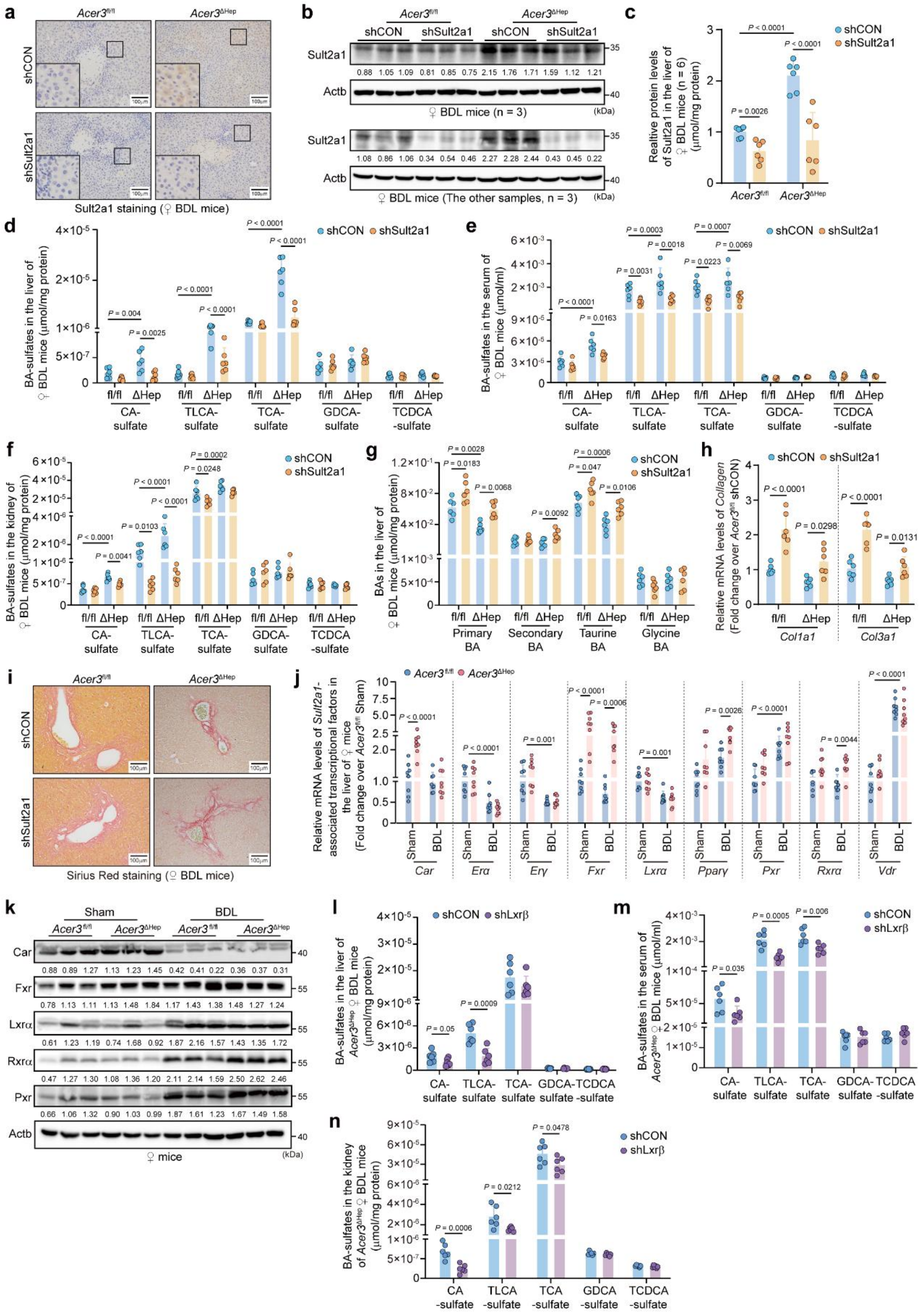

Figure S6. Profiling of Sult2a1-associated NR expression and *Sult2a1* or *Lxrβ* knockdown inhibits BA sulfation in the cholestatic liver of female mice.

(**a-i**) Evaluation of Sult2a1-catalyzed BA sulfation in the liver of *Acer3*$^{fl/fl}$ and *Acer3*$^{\Delta Hep}$ BDL female mice infected by shCON and shSult2a1 AAVs (n = 6). Sult2a1

staining in the liver sections (**a**). Sult2a1 immunoblot (**b**) and quantification of Sult2a1 protein in liver (**c**). Individual BA-sulfate species in the liver (**d**), serum (**e**), and kidney (**f**). Individual BA species (**g**). Collagen mRNA levels (**h**). Sirius Red staining in liver sections (**i**).

(**j** and **k**) Profiling of Sult2a1-associated nuclear receptor (NR) expression in the liver of *Acer3*$^{fl/fl}$ and *Acer3*$^{\Delta Hep}$ female mice subjected to sham operation or BDL (n = 8). The mRNA levels of Sult2a1-associated NRs in the liver (**j**). The protein levels of Sult2a1-associated NRs in the liver (**k**).

(**l-n**) Individual BA-sulfate species in the liver (**k**), serum (**l**), and kidney (**m**) of *Acer3*$^{\Delta Hep}$ female mice with or without liver X receptor β (Lxrβ) knockdown (n = 6).

Data are expressed as mean ± SD. Statistical significances were tested by the one-way ANOVA with Tukey's multiple comparisons test (**c-h**, **j**) and unpaired two-sided Student's *t*-test (**l-n**). Source data are provided as a Source Data file.

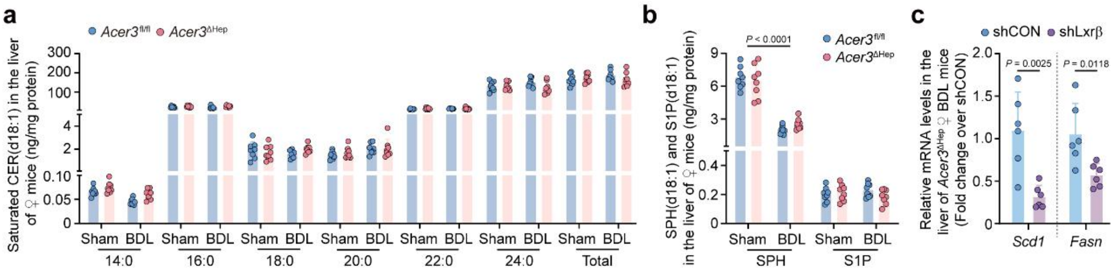

Figure S7. The impact of hepatocyte-specific *Acer3* ablation on saturated CERs, SPH, and S1P in female mice and the mRNA levels of *Scd1* and *Fasn* in the liver of *Acer3*$^{\Delta Hep}$ BDL female mice after *Lxrβ* knockdown.

(**a** and **b**) The levels of individual saturated ceramide (CER) (d18:1) species (**a**), SPH(d18:1) (**b**), and S1P(d18:1) (**b**) in the liver of *Acer3*$^{fl/fl}$ and *Acer3*$^{\Delta Hep}$ female mice subjected to BDL or sham operation (n = 8).

(**c**) The mRNA levels of *Scd1* and *Fasn* in the liver of *Acer3*$^{\Delta Hep}$ BDL female mice with or without *Lxrβ* knockdown (n = 6).

Data are expressed as mean ± SD. Statistical significances were tested by the one-way ANOVA with Tukey's multiple comparisons (**a**, **b**) and unpaired two-sided Student's *t*-test (**c**). Source data are provided as a Source Data file.

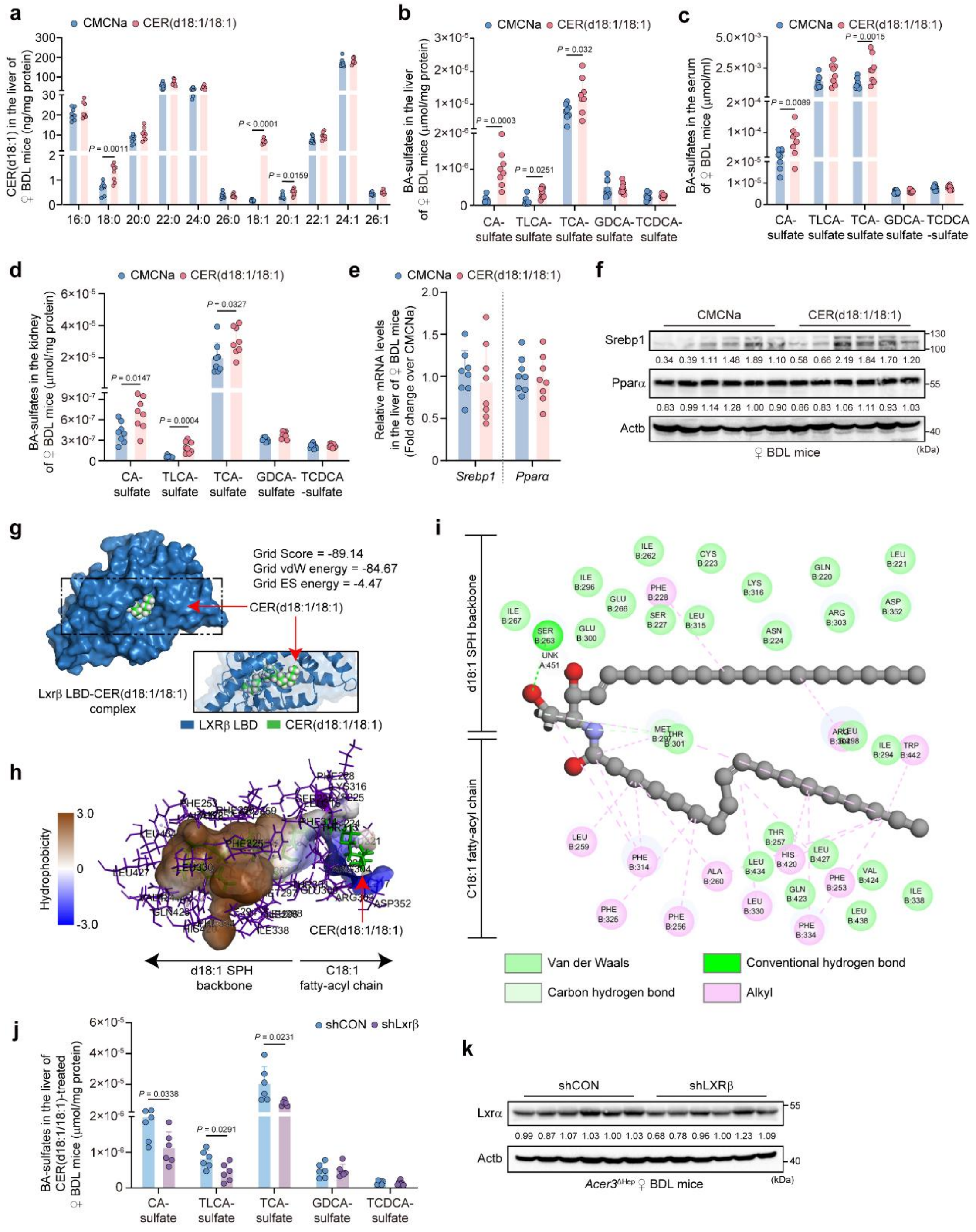

Figure S8. CER(d18:1/18:1) promotes Lxrβ-dependent BA sulfation in the cholestatic liver of female mice and the analysis of CER(d18:1/18:1)-Lxrβ interaction.

(**a**) CER(d18:1) in the liver of female BDL WT mice administrated with carboxymethylcellulose (CMC-Na) and CER(d18:1/18:1) (n = 8).

(**a-d**) Individual BA-sulfate species in the liver (**b**), serum (**c**), and kidney (**d**) of female BDL WT mice administrated with CMCNa and CER(d18:1/18:1) (n = 8).

(**e** and **f**) The mRNA levels (**e**) (n = 8) and protein levels (**f**) (n = 6) of sterol regulatory element binding protein 1 (Srebp1) and peroxisome proliferator-activated receptor alpha (Pparα) in the liver of female BDL WT mice administrated with CMCNa and CER(d18:1/18:1).

(**g**) Virtual 3D model of Lxrβ ligand binding domain (LBD)-CER(d18:1/18:1) complex.

(**h**) Hydrophobicity analysis of Lxrβ LBD-CER(d18:1/18:1) interaction.

(**i**) 2D diagram exhibiting the predicted amino acid residues of Lxrβ LBD interacting with CER(d18:1/18:1).

(**j**) Individual BA-sulfate species in the cholestatic liver of *Lxrβ*-knockdown and control female mice administrated with CER(d18:1/18:1) (n = 6).

(**k**) liver X receptor α (Lxrα) protein levels in the liver of $Acer3^{\Delta Hep}$ BDL female mice with or without *Lxrβ* knockdown (n = 6).

Data are expressed as mean ± SD. Statistical significances were tested by the unpaired two-sided Student's *t*-test (**a-e**, **j**). Source data are provided as a Source Data file.

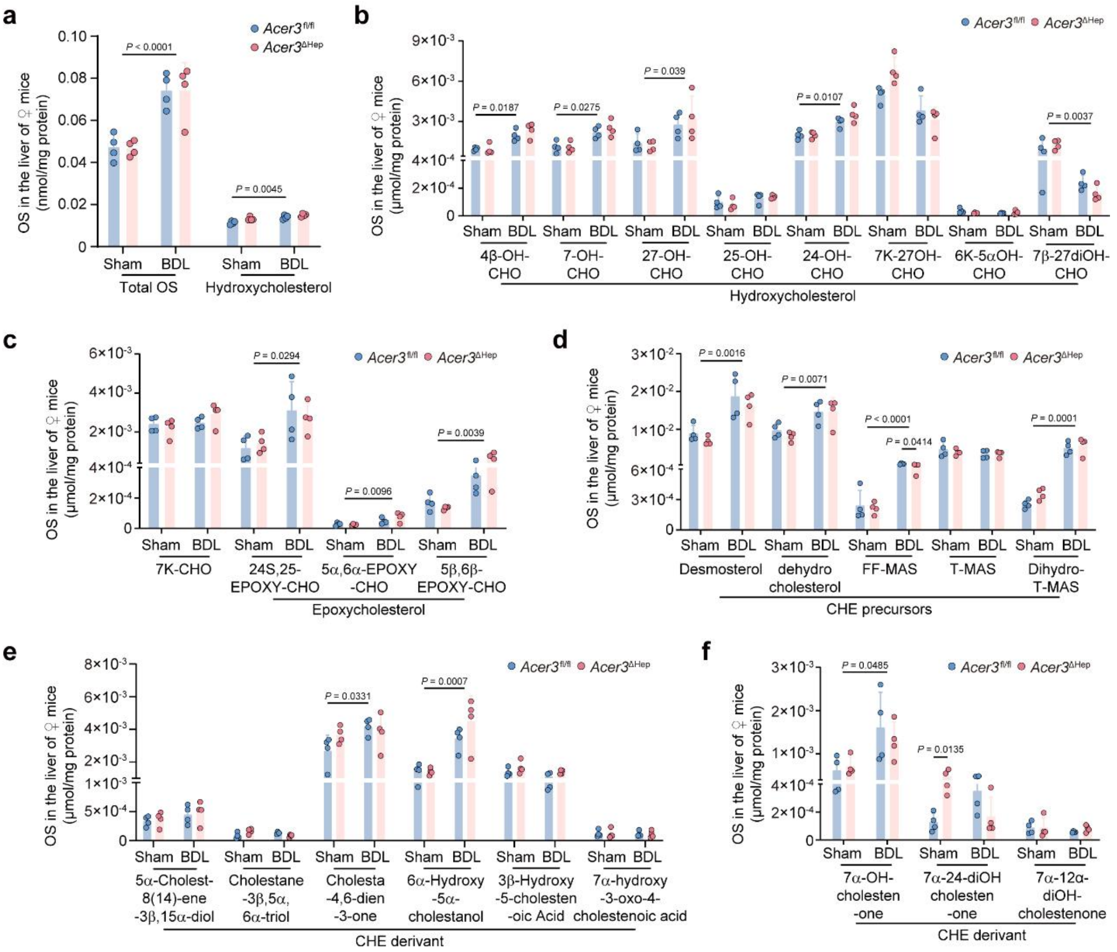

Figure S9. Hepatocyte-specific *Acer3* ablation has no effect on oxysterol in the liver of female mice.

(**a-f**) Targeted lipidomics of oxysterol (OS) in the liver of *Acer3*$^{fl/fl}$ and *Acer3*$^{\Delta Hep}$ female mice subjected to BDL or sham operation (n = 4). Total OS and hydroxycholesterol (**a**). Individual hydroxycholesterol species (**b**). Individual epoxycholesterol species (**c**). Individual cholesteryl ester (CHE) precursors species (**d**). Individual CHE derivates (**e** and **f**).

Data are expressed as mean ± SD. Statistical significances were tested by the one-way ANOVA with Tukey's multiple comparisons (**a-f**). Source data are provided as a Source Data file.

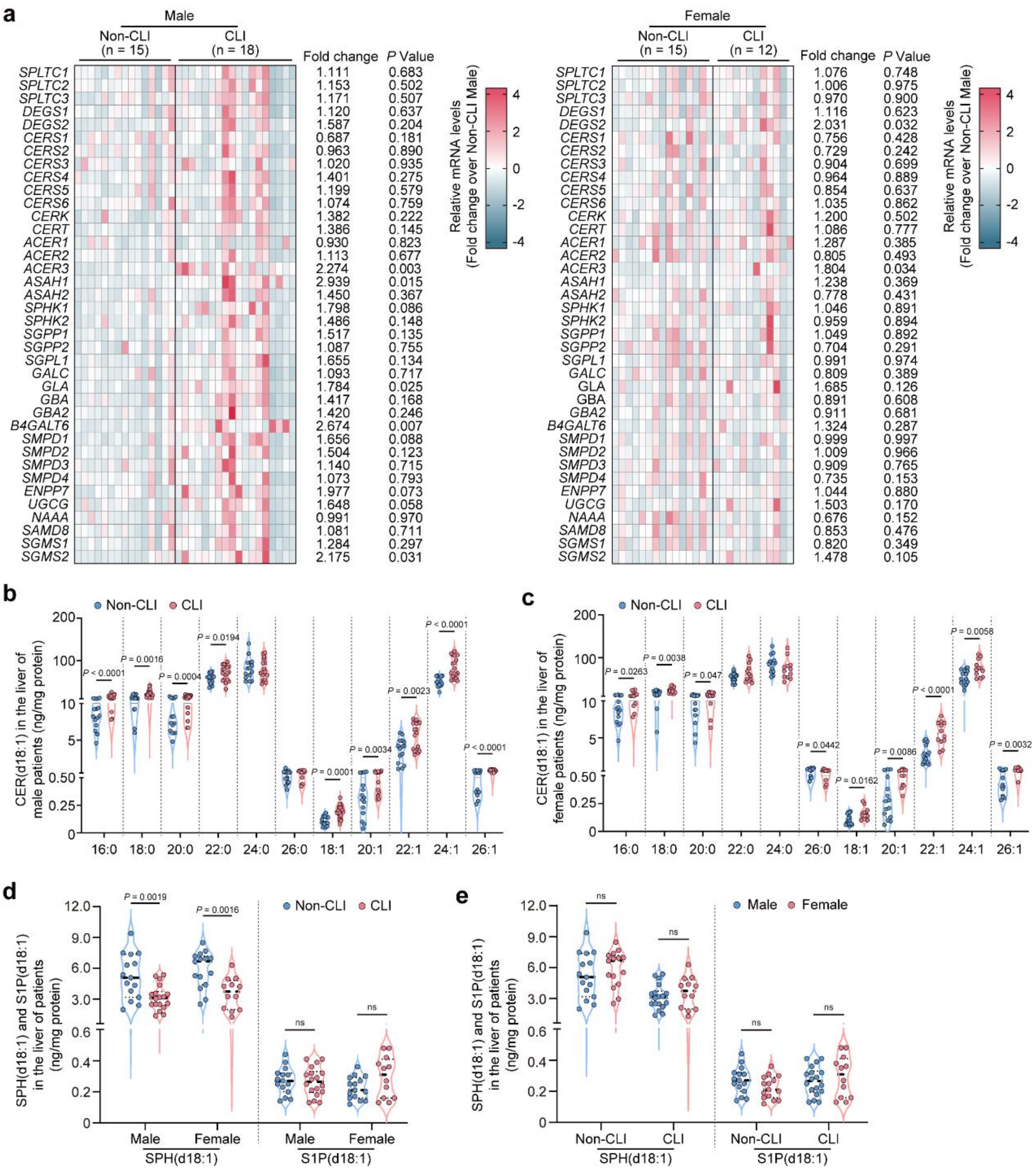

Figure S10. CLI induces the dysregulation of sphingolipid metabolism in the liver of patients.

(**a**) Heat maps of the mRNA levels of CER-metabolizing enzymes in the non-CLI and CLI liver tissues of male (left panel) and female (right panel) patients, fold change mean the ratio of non-CLI to CLI.

(**b**) CER(d18:1) levels in the collected liver tissues of male patients with non-CLI (n = 15) and CLI (n = 18).

(**c**) CER(d18:1) levels in the collected liver tissues of female patients with non-CLI (n = 15)and CLI (n = 12).

(**d** and **e**) The levels of SPH(d18:1) and S1P(d18:1) in the collected liver tissues of male and female patients with non-CLI and CLI, male patients with non-CLI (n = 15), male patients with CLI (n = 18), female patients with non-CLI (n = 15), female patients with CLI (n = 12).

Data are expressed as mean ± SD. Statistical significances were tested by the unpaired two-sided Student's *t*-test (**a-e**). Source data are provided as a Source Data file.

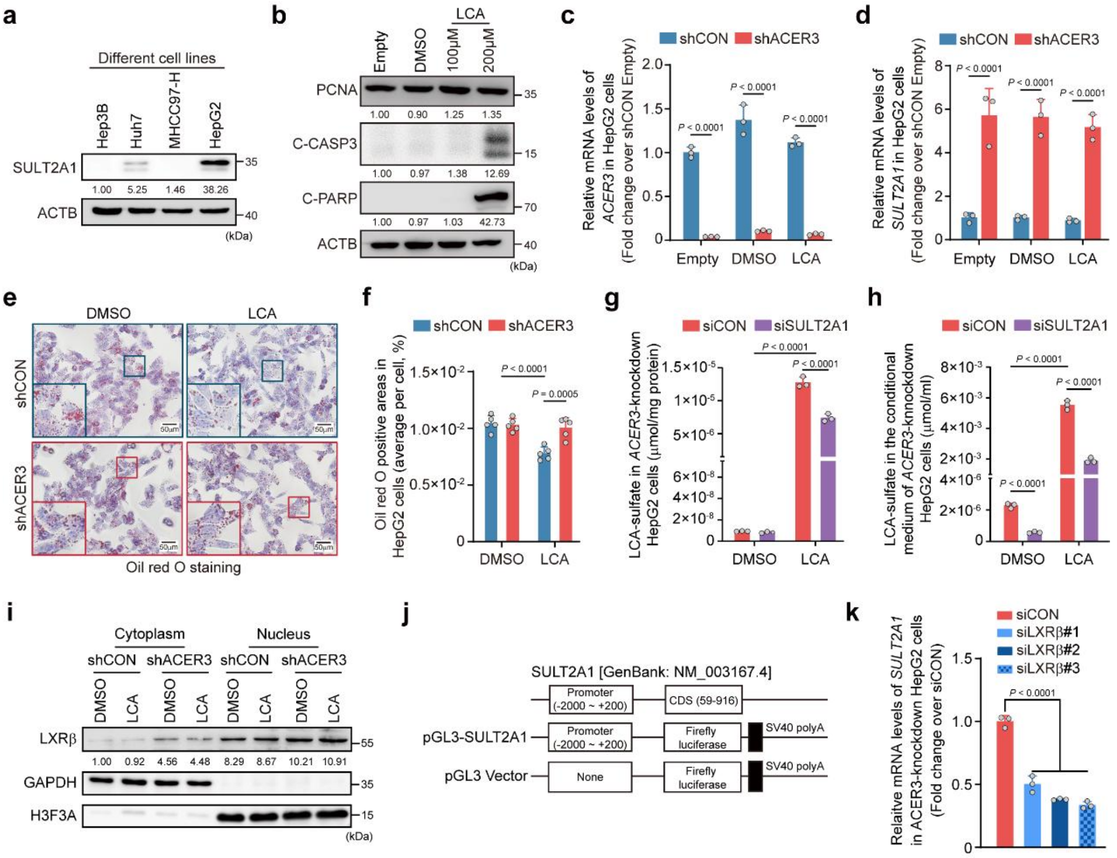

Figure S11. *ACER3* knockdown activates *LXRβ* to enhance SULT2A1-catalyzed BA sulfation to alleviate apoptosis and improve lipogenesis in LCA-treated HepG2 cells.

(**a**) Immunoblot of SULT2A1 in different human liver-derived cell lines.

(**b**) Immunoblot of proliferating cell nuclear antigen (PCNA), cleaved-caspase 3 (C-CASP3), and cleaved-poly ADP-ribose polymerase (C-PARP) in HepG2 cells treated with different concentrations of lithocholic acid (LCA).

(**c** and **d**) The mRNA levels of *ACER3* (**c**) and *SULT2A1* (**d**) in HepG2 cells transfected by shCON and shACER3 lentivirus following treatment of vehicle (transfection medium), dimethyl sulfoxide (DMSO), or 200 μM LCA.

(**e** and **f**) Oil red O staining of HepG2 cells with or without *ACER3* knockdown and LCA treatment (**e**) quantification of Oil red O positive areas (**f**).

(**g** and **h**) LCA-sulfate in *ACER3*-knockdown HepG2 cells (**g**) and the conditional medium of *ACER3*-knockdown HepG2 cells (**h**) with or without siSULT2A1#1 and LCA treatment.

(**i**) Immunoblot of LXRβ in the cytoplasm and nuclear extraction of HepG2 cells

infected by shCON and shACER3 lentivirus following treatment of DMSO, or LCA.

(**j**) Schematic diagram depicting the plasmid design of SULT2A1-luciferase.

(**k**) The mRNA levels of *SULT2A1* in *ACER3*-knockdown HepG2 cells transfected with *LXRβ* siRNA.

Data represent experiments from three independent experiments and are expressed as mean ± SD. Statistical significances were tested by the one-way ANOVA with Tukey's multiple comparisons (**c**, **d**, **f-h**, **k**). Source data are provided as a Source Data file.

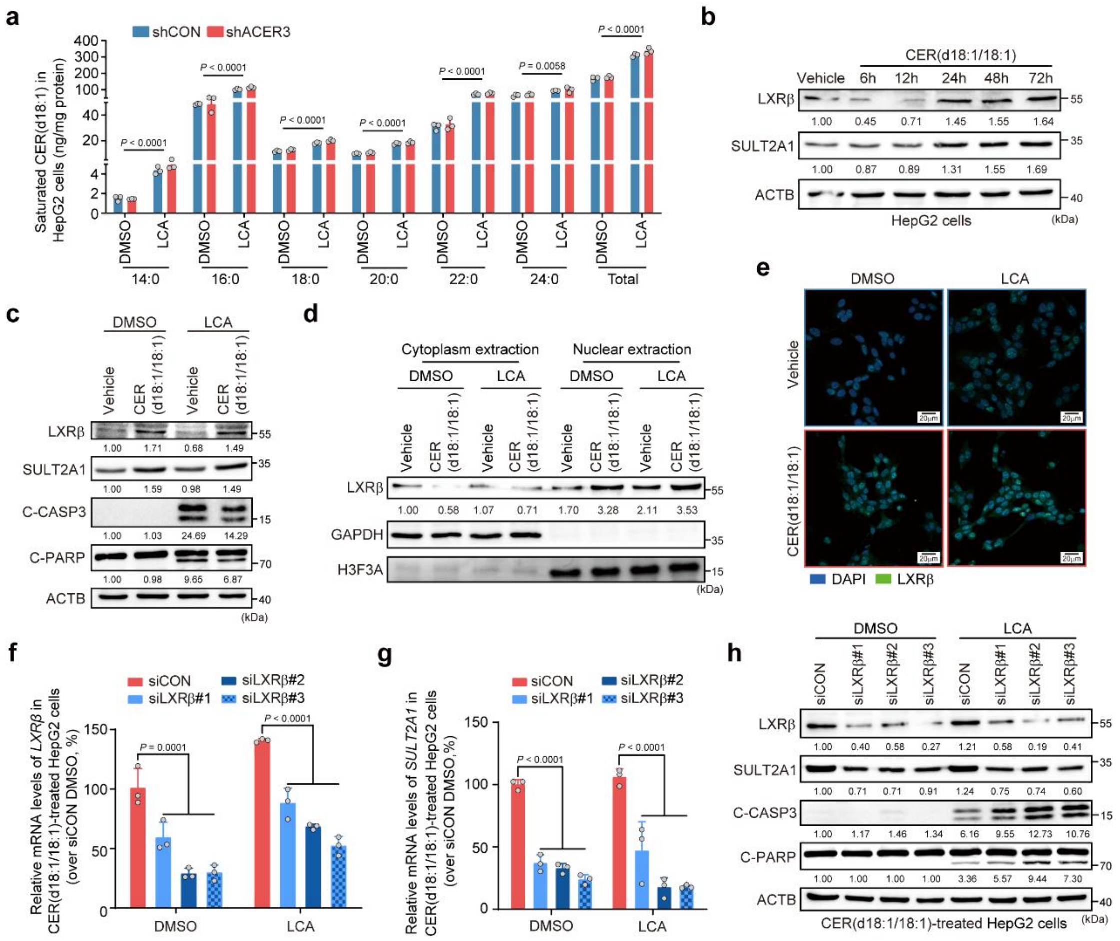

Figure S12. *ACER3* knockdown increases CER(d18:1/18:1) to upregulate *SULT2A1* through activating *LXRβ* in HepG2 cells.

(**a**) The levels of individual saturated CER(d18:1) species in HepG2 cells with or without *ACER3* knockdown and LCA.

(**b**) Immunoblot of LXRβ and SULT2A1 in HepG2 cells treated with 5 μM CER(d18:1/18:1) for 6, 12, 24, 48, and 72 hours.

(**c**) Immunoblot of LXRβ, SULT2A1, C-CASP3, and C-PARP in HepG2 cells treated with or without CER(d18:1/18:1) and LCA.

(**d** and **e**) Immunoblot of LXRβ in the cytoplasm and nuclear extraction (**d**) and immunofluorescence of LXRβ cells (**e**) of HepG2 treated with or without CER(d18:1/18:1) and LCA.

(**f** and **g**) The mRNA levels of *LXRβ* (**f**) and *SULT2A1* (**g**) in CER(d18:1/18:1)-treated *LXRβ*-knockdown HepG2 cells subjected to DMSO and LCA treatment.

(**h**) Immunoblot of LXRβ, SULT2A1, C-CASP3, and C-PARP in CER(d18:1/18:1)-

treated *LXRβ*-knockdown HepG2 cells subjected to DMSO and LCA treatment.

Data represent experiments from three independent experiments and are expressed as mean ± SD. Statistical significances were tested by the one-way ANOVA with Tukey's multiple comparisons (**a**, **f**, **g**). Source data are provided as a Source Data file.

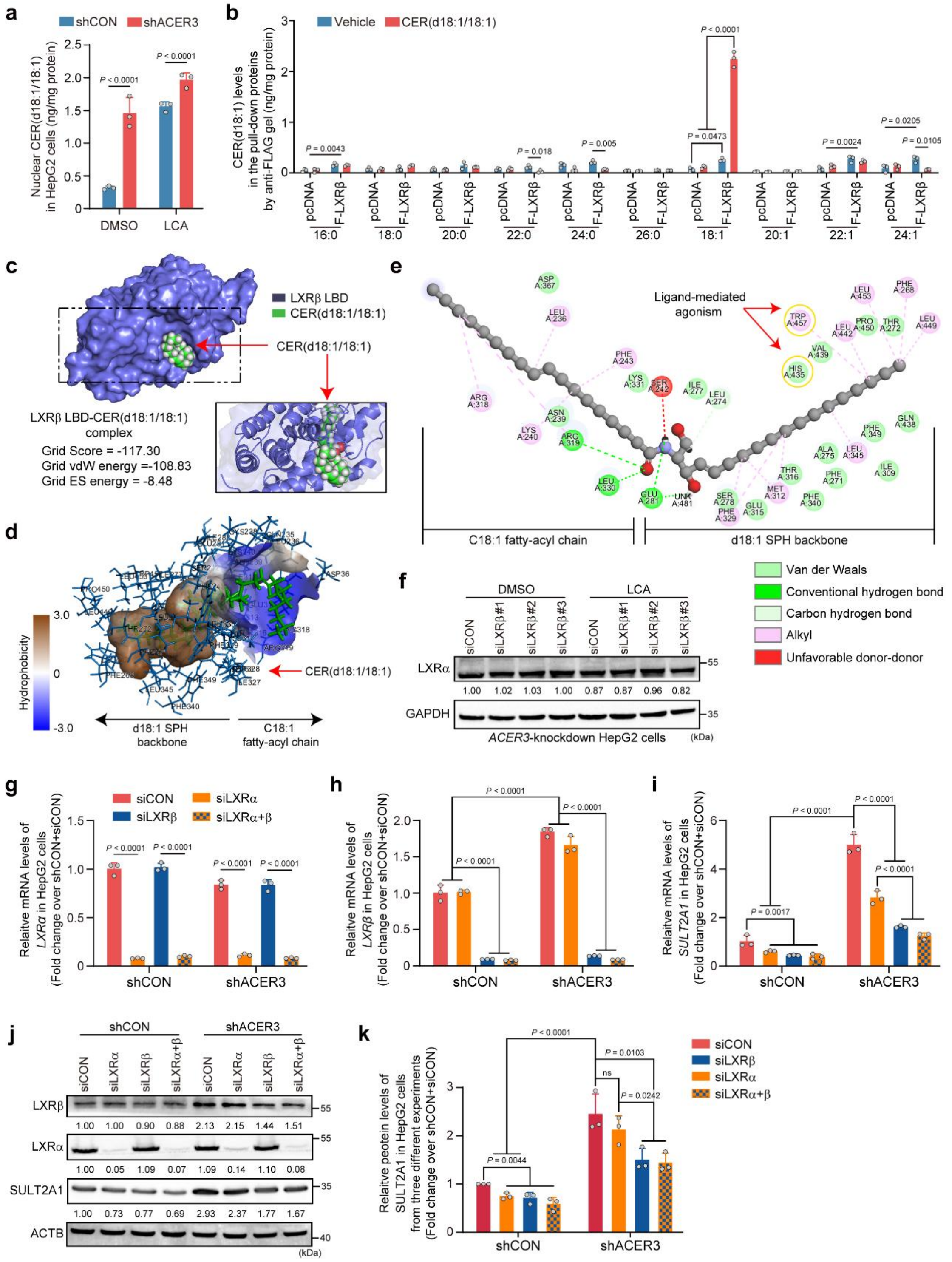

Figure S13. CER(d18:1/18:1) binds with LXRβ in HepG2 cells.

(**a**) The nuclear levels of CER(d18:1/18:1) in HepG2 with or without *ACER3* knockdown and LCA treatment.

(**b**) The levels of individual CER(d18:1) species in immunoprecipitated LXRβ proteins from HepG2 cells treated with or without CER(d18:1/18:1).

(**c**) Virtual 3D model of human LXRβ LBD-CER(d18:1/18:1) complex.

(**d**) Hydrophobicity of LXRβ LBD -CER(d18:1/18:1) interaction.

(**e**) A 2D diagram exhibiting the predicted amino acid residues of LXRβ LBD interacting with CER(d18:1/18:1).

(**f**) *LXRα* protein levels in *ACER3*-knockdown HepG2 cells following transfection of siLXRα and siLXRβ with or without LCA treatment.

(**g-i**) The mRNA levels of *LXRα* (**g**), *LXRβ* (**h**), and *SULT2A1* (**i**) in HepG2 cells infected by shCON and shACER3 lentivirus following transfection of siLXRα and siLXRβ#2.

(**j**) The protein levels of *LXRα*, *LXRβ*, and *SULT2A1* in HepG2 cells infected by shCON and shACER3 lentivirus following transfection of siLXRα and siLXRβ#2.

(**k**) Quantification of SULT2A1 protein in the control and *ACER3*-knockdown HepG2 cells following transfection of siLXRα and siLXRβ#2 from three independent experiments.

Data represent experiments from three independent experiments and are expressed as mean ± SD. Statistical significances were tested by the one-way ANOVA with Tukey's multiple comparisons (**a**, **b**, **g-i**, **k**). Source data are provided as a Source Data file.

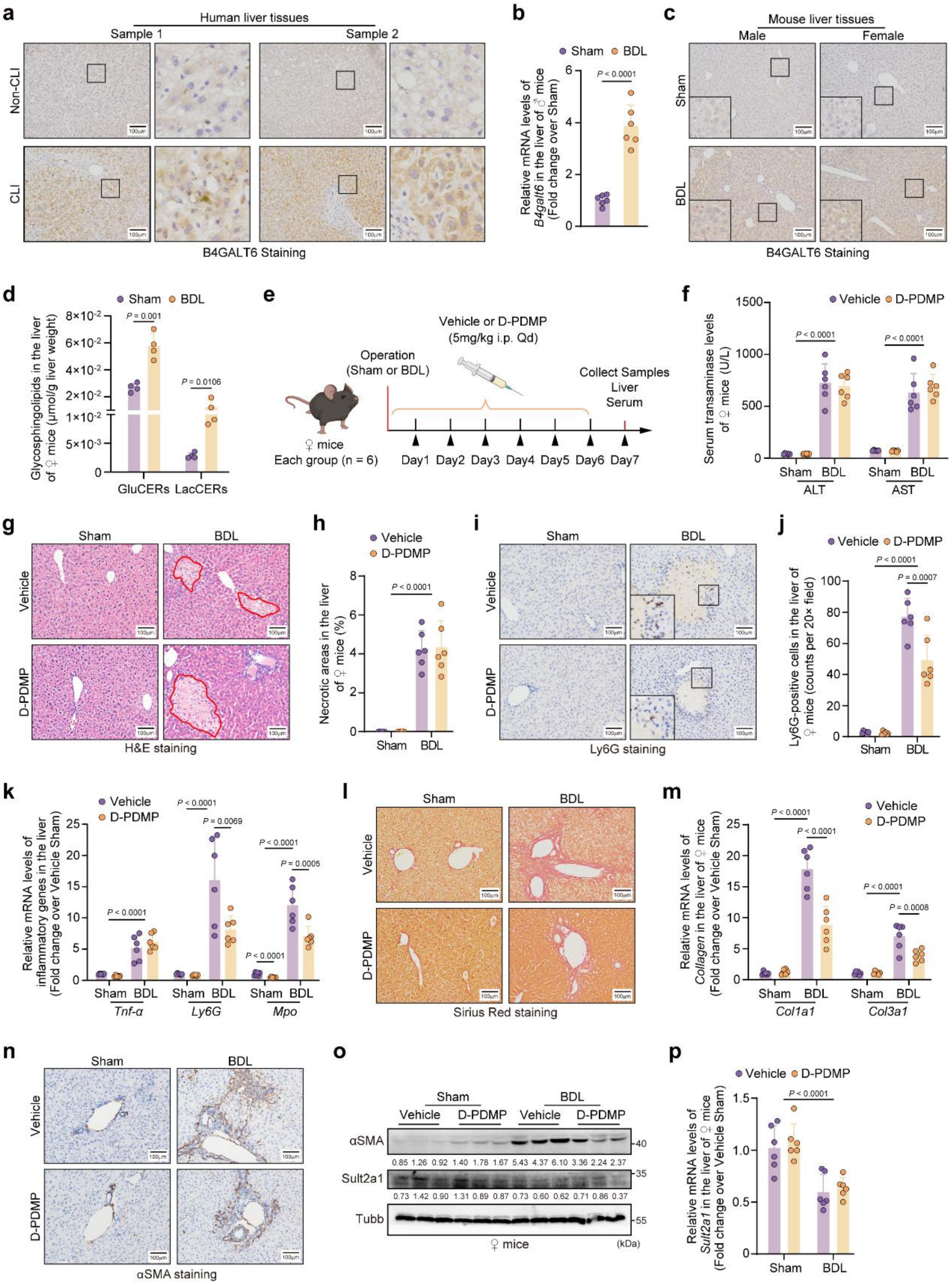

Figure S14. Cholestasis upregulates B4GALT6/B4galt6 and pharmacological inhibition of B4galt6 alleviates BDL-induced inflammation and fibrosis without affecting Sult2a1 in female mice.

(**a**) Beta-1,4-galactosyltransferase 6 (B4GALT6) staining in liver sections of patients with non-CLI and CLI (n =30).

(**b**) B4galt6 mRNA levels in the liver of male C57BL/6J WT mice under BDL and

sham conditions (n = 6).

(**c**) B4galt6 staining in the liver of WT mice under BDL and sham conditions.

(**d**) Glucosylceramides (GluCERs) and lactosylceramides (LacCERs) in the liver of female WT mice under BDL and sham conditions (n = 4).

(**e-p**) Examination of CLI in WT female mice administrated with a vehicle (10% ETOH, 40% PEG300, 5% Tween-80, and 45% saline) and B4galt6 inhibitor, d-threo-1-phenyl-2-decanoylamino-3-morpholino-1-propanol (D-PDMP) (n = 6). Schematic diagram of D-PDMP treatment in WT female mice. Created in BioRender. Liao, L. (2025), https://BioRender.com/l76t693. (**e**). Serum transaminase levels (**f**). H&E staining with the circle areas and red arrows indicating necrotic foci (**g**) and quantification of necrotic areas (**h**) in liver sections. Ly6G staining (**i**) and quantification of Ly6G-positive cells in liver sections (**j**). The mRNA levels of inflammatory genes in the liver (**k**). Sirius Red staining in liver sections (**l**). Collagen mRNA levels (**m**). αSMA staining in liver sections (**n**). Immunoblot of αSMA and Sult2a1 (**o**). *Sult2a1* mRNA levels (**p**).

Data are expressed as mean ± SD. Statistical significances were tested by the unpaired two-sided Student's *t*-test (**b**, **d**) and one-way ANOVA with Tukey's multiple comparisons (**f**, **h**, **j**, **k**, **m**, **p**). Source data are provided as a Source Data file.

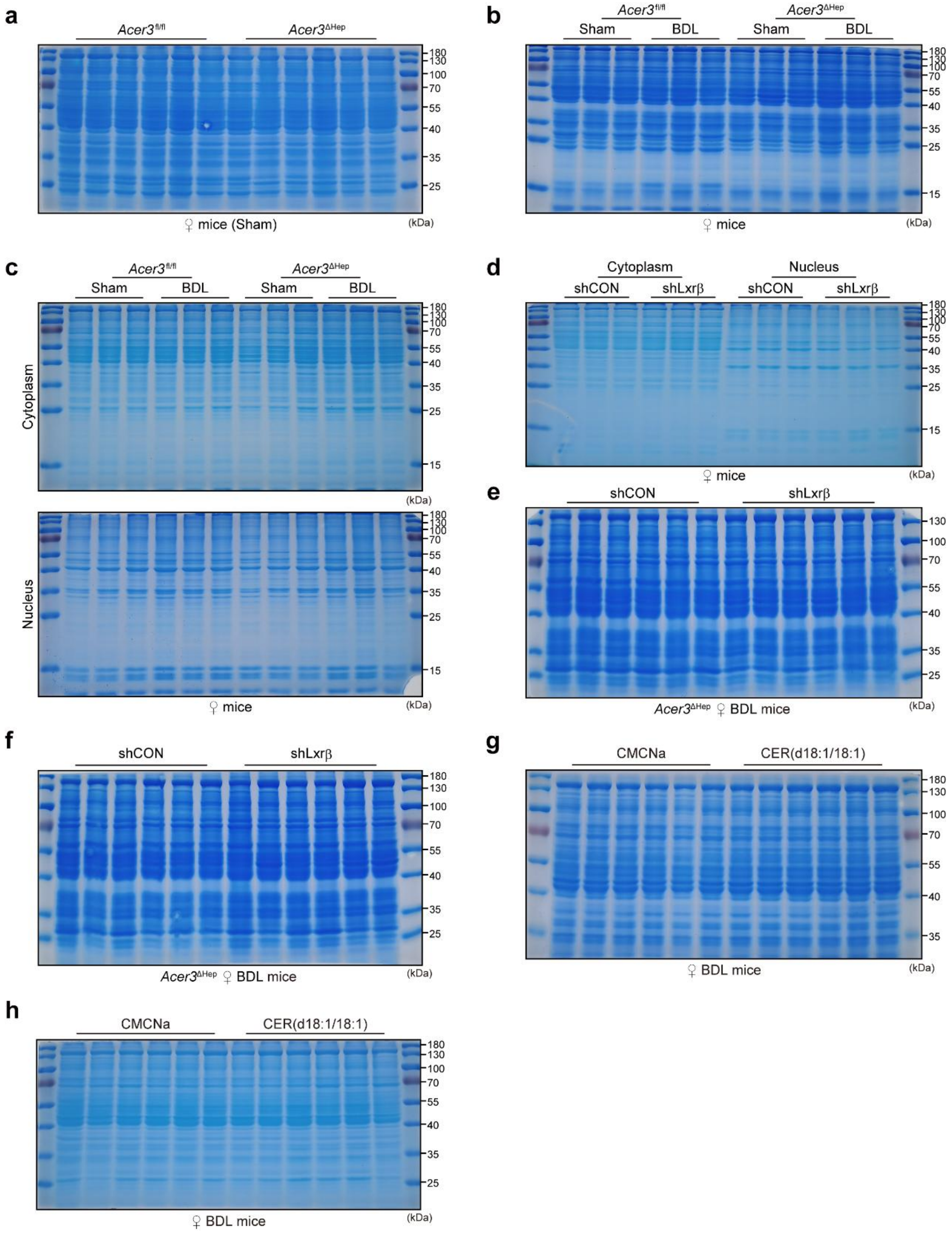

Figure S15. Coomassie blue staining of gels from western blot.

(**a**) Coomassie blue staining of Lxrβ immunoblot for the liver protein of *Acer3*$^{fl/fl}$ and *Acer3*$^{\Delta Hep}$ female mice with sham operation, corresponding to Figure 3b.

(**b**) Coomassie blue staining of Lxrβ immunoblot for the liver protein of *Acer3*$^{fl/fl}$ and *Acer3*$^{\Delta Hep}$ female mice under sham and BDL conditions, corresponding to Figure 3c.

(**c**) Coomassie blue staining of immunoblot of cytoplasmic and nuclear Lxrβ for the

liver protein of *Acer3*$^{fl/fl}$ and *Acer3*$^{\Delta Hep}$ female mice under sham and BDL conditions, corresponding to Figure 3f.

(**c**) Coomassie blue staining of immunoblot for cytoplasmic and nuclear Lxrβ for the liver protein of *Acer3*$^{\Delta Hep}$ female BDL mice with or without *Lxrβ* knockdown, corresponding to Figure 3h.

(**e**) Coomassie blue staining of immunoblot of Lxrβ and Sult2a1 for the liver protein of *Acer3*$^{\Delta Hep}$ female BDL mice with or without *Lxrβ* knockdown, corresponding to Figure 3i.

(**f**) Coomassie blue staining of αSMA immunoblot for the liver protein of *Acer3*$^{\Delta Hep}$ female BDL mice with or without *Lxrβ* knockdown, corresponding to Figure 3v.

(**g**) Coomassie blue staining of αSMA immunoblot for the liver protein of WT BDL female mice administrated with CMCNa and CER(d18:1/18:1), corresponding to Figure 5j.

(**h**) Coomassie blue staining of immunoblot of Lxrβ and Sult2a1 for the liver protein of WT BDL female mice administrated with CMCNa and CER(d18:1/18:1), corresponding to Figure 5n.
