# Supplementary material for: Targeting the ceramidase ACER3 attenuates cholestasis in mice by mitigating bile acid overload via unsaturated ceramide-mediated LXRβ signaling transduction
Source: Nat Commun. 2025 Mar 2;16:2112. doi: 10.1038/s41467-025-57330-7 (PMC11873283; doi:10.1038/s41467-025-57330-7)
Supplement: Supplementary file 2 — Reporting Summary [file 41467_2025_57330_MOESM2_ESM.pdf]

nature portfolio

Corresponding author(s): Kai Wang

Last updated by author(s): Jan 30, 2025

Reporting Summary

Nature Portfolio wishes to improve the reproducibility of the work that we publish. This form provides structure for consistency and transparency in reporting. For further information on Nature Portfolio policies, see our Editorial Policies and the Editorial Policy Checklist.

Statistics

For all statistical analyses, confirm that the following items are present in the figure legend, table legend, main text, or Methods section.

n/a	Confirmed	
☐	☒	The exact sample size (*n*) for each experimental group/condition, given as a discrete number and unit of measurement
☐	☒	A statement on whether measurements were taken from distinct samples or whether the same sample was measured repeatedly
☐	☒	The statistical test(s) used AND whether they are one- or two-sided<br>*Only common tests should be described solely by name; describe more complex techniques in the Methods section.*
☒	☐	A description of all covariates tested
☐	☒	A description of any assumptions or corrections, such as tests of normality and adjustment for multiple comparisons
☐	☒	A full description of the statistical parameters including central tendency (e.g. means) or other basic estimates (e.g. regression coefficient) AND variation (e.g. standard deviation) or associated estimates of uncertainty (e.g. confidence intervals)
☐	☒	For null hypothesis testing, the test statistic (e.g. *F*, *t*, *r*) with confidence intervals, effect sizes, degrees of freedom and *P* value noted<br>*Give P values as exact values whenever suitable.*
☒	☐	For Bayesian analysis, information on the choice of priors and Markov chain Monte Carlo settings
☒	☐	For hierarchical and complex designs, identification of the appropriate level for tests and full reporting of outcomes
☒	☐	Estimates of effect sizes (e.g. Cohen's *d*, Pearson's *r*), indicating how they were calculated

Our web collection on statistics for biologists contains articles on many of the points above.

Software and code

Policy information about availability of computer code

Data collection

1. Western blot developed images were acquired using Touch Imager System N30447 (e-BLOT Life Science, Shanghai, China).
2. qRT-PCR data were collected by LightCycler 480 (Roche, Auckland, New Zealand).
3. Staining images were acquired using Intelligently Designed Microscope (Olympus, Shinjuku-ku, Tokyo, Japan).
4. Immunoflorescence staining images in the confocal dishes were acquired using Laser Scanning Confocal Microscope LSM 980 (ZEISS, Oberkochen, Germany).
5. Luciferase activity was determined by the Gen5 (Biotek, USA).
6.Sequenceing data were acquired using Illumina NovaSeq6000 platform.
7.Bile acids were determined on an Exion AD30-UPLC coupled with Sciex QTRAP 6500 Plus under electrospray ionization mode or prelude SPLC coupled with TSQ Quantiva system (Thermo Fisher Scientific, Waltham, MA, USA).
8. Untargeted lipidomics were analyzed using Thermo Fisher Scientific Vanquish Flex ultra-high-performance liquid chromatography (UHPLC) equipped with Thermo Fisher Scientific Orbitrap Fusion Tribrid High-Resolution Mass Spectrometer (Thermo Fisher Scientific, Waltham, MA, USA).
9. Targeted lipidomics of ceramides were determined by LC-MS/MS performed on prelude SPLC coupled with TSQ Quantiva system.
10. Targeted lipidomic of oxysterol were determined by Thermofisher U3000 DGLC coupled to Sciex QTRAP 6500 Plus/Shimadzu 40X3B-UPLC coupled to Sciex QTRAP 6500 Plus.
11. Surface plasmon resonance was performed using the PlexArray HT A100 (Plexera; Seattle, USA).

Data analysis

1. Images analysis were done using Image Pro Plus (version 6.0).
2. All the statistical analyses of the data were done using Prism-GraphPad version 9.0.
3. The mouse genomic and genetic information was obtained from the National Center for Biotechnology Information database. Expression levels of mRNA were evaluated using StringTie software (v1.3.44d). The analysis of differentially expressed genes (DEGs) were performed

using DESeq2 (version 1.34.0).
4. The identification of lipid molecular species was conducted by Lipid Search software (Thermo Fisher Scientific, Waltham, MA, USA).
5. Molecular docking was accomplished by UCSF Chimera (version 1.17.3) and DOCK 6.9.
6. Surface plasmon resonance was analyzed with BIA evaluation software (version 4.1).
7. R version 4.3.2 was used to draw the graph.

For manuscripts utilizing custom algorithms or software that are central to the research but not yet described in published literature, software must be made available to editors and reviewers. We strongly encourage code deposition in a community repository (e.g. GitHub). See the Nature Portfolio guidelines for submitting code & software for further information.

Data

Policy information about availability of data

All manuscripts must include a data availability statement. This statement should provide the following information, where applicable:

- Accession codes, unique identifiers, or web links for publicly available datasets
- A description of any restrictions on data availability
- For clinical datasets or third party data, please ensure that the statement adheres to our policy

The RNA-seq data generated in this study have been deposited in the Sequence Read Archive (SRA) database under accession codes PRJNA1182846 (https://www.ncbi.nlm.nih.gov/sra/PRJNA1182846) and PRJNA1184181 (https://www.ncbi.nlm.nih.gov/sra/PRJNA1184181). Deposited data are publicly available. The processed data of RNA-seq are available within source data. RNA-seq data of human liver tissues were obtained by GTEx (Genotype-Tissue Expression) database (https://www.gtexportal.org/). The raw data of the mouse hepatic lipidome have been deposited in the MetaboLights database under accession code MTBLS12198. Utilizing the hTFtarget, ChIP-Atlas, GTRD, ENCODE, and JASPAR databases, transcription factor prediction was accomplished by the online predicted tool (https://jingle.shinyapps.io/TF_Target_Finder/). The remaining data in this study are available within the manuscript or Supplementary Data. Source data are provided with this paper.

Research involving human participants, their data, or biological material

Policy information about studies with human participants or human data. See also policy information about sex, gender (identity/presentation), and sexual orientation and race, ethnicity and racism.

Reporting on sex and gender

Sex was a critical factor in our study design, and both male and female C57BL/6J mice were included. We observed that Acer3 ablation significantly attenuated CLI in female mice, while the effect in male mice was minimal. Consequently, we focused on female mice to further investigate the mechanism of ACER3 regulation in CLI. In the incorporating sex-based analyses for patients, patient samples were divided by sex, and comparisons were made for hepatic CER content, related enzyme expression levels, and SULT2A1 across the groups. Additionally, we utilized an online dataset to analyze the expression of CER-related enzymes and SULT2A1 in healthy male and female liver tissues. Detailed baseline characteristics, including sex-specific data, are provided in the Supplementary Data. All sex-related information was derived from the patient's medical records.

Reporting on race, ethnicity, or other socially relevant groupings

All of patients in our study were Asian. The study did not considered other socially relevant groupings.

Population characteristics

The characteristics of patients were illustrated in Table S1 and S2.

Recruitment

Patient samples were collected from patients with CLI caused by bile duct obstruction and without CLI who underwent hepatectomy at the Division of Hepatobiliopancreatic Surgery, Department of General Surgery, Nanfang Hospital, Southern Medical University. Samples were utilized in this study per availability. There was no active recruitment of patients for this study.

Ethics oversight

The study was approved by the Medical Ethics Committee of Nanfang Hospital of Southern Medical University under ethical (ID NFEC-2021-356).

Note that full information on the approval of the study protocol must also be provided in the manuscript.

Field-specific reporting

Please select the one below that is the best fit for your research. If you are not sure, read the appropriate sections before making your selection.

☒ Life sciences ☐ Behavioural & social sciences ☐ Ecological, evolutionary & environmental sciences

For a reference copy of the document with all sections, see nature.com/documents/nr-reporting-summary-flat.pdf

Life sciences study design

All studies must disclose on these points even when the disclosure is negative.

Sample size

We decide sample size based on our working experiences in this field, as reported in our previous publications (PMID: 7998020, PMID: 32738398, PMID: 6965144, and PMID: 4823937). For in vitro experiments, a minimum of 3 independent samples were used. For in vivo studies, at least 4 mice per group were anticipated in most of the experiments.

Data exclusions	All the animals were housed in animal facility for one week before operation to exclude any unhealthy mice.
Replication	Three independent experiments verify the reproducibility of the in vitro experimental findings. All the animal experiments were done at least twice, and each group had at least 5 mice. The replication of In vivo experimental findings were validated using at least 3 different biological samples each groups. All attempts at replication were successful.
Randomization	Sample allocation to each experimental group was randomized. The littermate mice were allocated randomly to the control group and the experimental group via a table of random numbers.
Blinding	To avoid observer-expectation bias, sample acquisition, experimental operation, and final data analysis were performed by different people.

Reporting for specific materials, systems and methods

We require information from authors about some types of materials, experimental systems and methods used in many studies. Here, indicate whether each material, system or method listed is relevant to your study. If you are not sure if a list item applies to your research, read the appropriate section before selecting a response.

Materials & experimental systems

n/a	Involved in the study	
☐	☒	Antibodies
☐	☒	Eukaryotic cell lines
☒	☐	Palaeontology and archaeology
☐	☒	Animals and other organisms
☒	☐	Clinical data
☒	☐	Dual use research of concern
☒	☐	Plants

Methods

n/a	Involved in the study	
☒	☐	ChIP-seq
☒	☐	Flow cytometry
☒	☐	MRI-based neuroimaging

Antibodies

Antibodies used

Anti-ACER3 Rabbit mAb, Sigma-Aldrich, Cat: #HPA070087 (1:200)
Anti-αSMA Rabbit mAb, Cell Signaling Technology, Cat: #19245S (1:1000)
Anti-SULT2A1 Rabbit mAb, Abcam, Cat: #ab194113 (1:1000)
Anti-LXRβ Rabbit pAb, Abcam, Cat: #ab28479 (1:1000)
Anti-LXRα Rabbit mAb, Abcam, Cat: #ab176323 (1:1000)
Anti-FXR Mouse mAb, Cell Signaling Technology, Cat: #72105S (1:1000)
Anti-PXR Rabbit pAb, Abcam, Cat: #ab192579 (1:1000)
Anti-CAR Rabbit pAb, Abcam, Cat: #ab186869 (1:1000)
Anti-RXRα Rabbit mAb, Abcam, Cat: #ab125001 (1:1000)
Anti-LY6G Rabbit mAb, Abcam, Cat: #ab238132 (1:2000)
Anti-ALB Mouse mAb, Proteintech, Cat: #16475-1-AP (1:200)
Anti-PCNA Rabbit mAb, Cell Signaling Technology, Cat: #13110S (1:1000)
Anti-B4GALT6 Rabbit mAb, Proteintech, Cat: #20148-1-AP (1:200)
Anti-SREBP1 Rabbit mAb, Abcam, Cat: #ab313881 (1:1000)
Anti-PPARα Rabbit mAb, Abcam, Cat: #ab314112 (1:1000)
Anti-DEGS2 Rabbit pAb, Thermo Fisher Scientific, Cat: #PA5-24082 (1:1000)
Anti-SMPD3 Mouse pAb, Thermo Fisher Scientific, Cat: #PA5-117447 (1:500)
Anti-GLA Mouse mAb, Proteintech, Cat: #66121-1-IG (1:1000)
Anti-CERS3 Rabbit mAb, Thermo Fisher Scientific, Cat: #PA5-113105 (1:1000)
Anti-Cleaved-caspase 3 Rabbit mAb, Cell Signaling Technology, Cat: #9664S (1:1000)
Anti-Cleaved-PARP Rabbit mAb, Cell Signaling Technology, Cat: #5625S (1:1000)
Anti-Histone H3 Rabbit pAb, Abcam, Cat: #ab1791 (1:1000)
Anti-FLAG Rabbit mAb, Abcam, Cat: #ab205606 (1:1000)
Anti-GAPDH Mouse mAb, Abcam, Cat: #ab8245 (1:2000)
Anti-β-Tubulin Rabbit mAb, Abcam, Cat: #ab68193 (1:2000)
Anti-β-Actin Rabbit mAb, Cell Signaling Technology, Cat: #4970S (1:2000)

Validation

The antibodies are validated by the manufacturers, as appears on the manufacturers' websites.
Anti-ACER3 Rabbit mAb: IHC(Human), validated by Sigma-Aldrich.
Anti-αSMA Rabbit mAb: WB/IP/IHC/IF(Human/Mouse/Rat/Hamster/Monkey), validated in 992 publications.
Anti-SULT2A1 Rabbit mAb: FC/IHC/WB/IF(Human/Mouse/Rat), validated in 6 publications.
Anti-LXRβ Rabbit pAb: WB/IF(Human/Mouse/Rat), validated in 18 publications.
Anti-LXRα Rabbit mAb: WB(Human/Mouse/Rat/Sheep), validated in 57 publications.
Anti-FXR Mouse mAb: WB/IP(Human/Mouse), validated in 17 publications.
Anti-PXR Rabbit pAb: WB(Rat/Mouse), validated in 15 publications.
Anti-CAR Rabbit pAb: WB/IF/IHC(Human/Mouse/Rat), validated in 6 publications.
Anti-RXRα Rabbit mAb: WB/IP/IF(Human/Mouse/Rat), validated in 27 publications.
Anti-LY6G Rabbit mAb: FC/IHC(Mouse), validated in 45 publications.
Anti-ALB Mouse mAb: WB/IF/IHC/IP/ELISA(Human/Mouse/Rat), validated in 156 publications.

Anti-PCNA Rabbit mAb: WB/IP/IHC/IF(Human/Mouse/Rat/Monkey), validated in 685 publications.
Anti-B4GALT6 Rabbit mAb: WB/IF/IHC/ELISA(Human/Mouse/Rat), validated in 3 publications.
Anti-SREBP1 Rabbit mAb: WB(Human/Mouse), validated in 2 publications.
Anti-PPARα Rabbit mAb: WB(Human/Rat), validated by Abcam.
Anti-DEGS2 Rabbit pAb: WB/IHC(Human/Mouse), validated in 2 publications.
Anti-SMPD3 Mouse pAb: WB(Human/Mouse), validated by Thermo Fisher Scientific.
Anti-GLA Mouse mAb: WB/IF/IHC/ELISA(Human), validated in 2 publications.
Anti-CERS3 Rabbit mAb: WB/ELISA(Human/Mouse), validated by Thermo Fisher Scientific.
Anti-Cleaved-caspase 3 Rabbit mAb: WB/IP/IHC/IF(Human/Mouse/Rat/Monkey), validated in 6514 publications.
Anti-Cleaved-PARP Rabbit mAb: WB/IP/IHC/IF(Human/Monkey), validated in 1788 publications.
Anti-Histone H3 Rabbit pAb: WB/IF/IHC/IP(Human/Mouse/Rat), validated in 4739 publications.
Anti-FLAG Rabbit mAb, Abcam: WB/IF/IHC/IP/FC(species independent), validated in 115 publications.
Anti-GAPDH Mouse mAb: WB/IF(Human/Mouse/Rat), validated in 5150 publications.
Anti-β-Tubulin Rabbit mAb: WB/IF/IHC/FC(Human/Mouse/Rat), validated in 190 publications.
Anti-β-Actin Rabbit mAb: WB/IF/IHC(Human/Mouse/Rat/Bovine/Pig), validated in 6879 publications.

Eukaryotic cell lines

Policy information about cell lines and Sex and Gender in Research

Cell line source(s)
All of cell lines come from Shanghai Cell Bank of the Academy of Chinese Sciences and Liver Cancer Institute, including HepG2 (ID: TCHu72), Huh-7 (ID: TCHu182), Hep3B (ID: TCHu106), MHCC97-H (ID: SCSP-5092).

Authentication
All of cell lines were purchased from Shanghai Cell Bank of the Academy of Chinese Sciences and Liver Cancer Institute. These cell lines were authenticated through Short Tandem Repeat (STR) analysis to verify their identity and uniqueness.

Mycoplasma contamination
Cell lines were tested for Mycoplasma contamination.

Commonly misidentified lines (See ICLAC register)
The study did not used the commonly misidentified lines.

Animals and other research organisms

Policy information about studies involving animals; ARRIVE guidelines recommended for reporting animal research, and Sex and Gender in Research

Laboratory animals
The study used six-to-eight-week-old female and male mice with C57BL/6J genetic background. Acer3 deficient and their control female and male mice were breed and reared at the animal facilities of Southern Medical University.

Wild animals
The study did not involve wild animals.

Reporting on sex
Sex was a critical factor in our study design, and both male and female C57BL/6J mice were included. We observed that Acer3 ablation significantly attenuated CLI in female mice, while the effect in male mice was minimal. Consequently, we focused on female mice to further investigate the mechanism of ACER3 regulation in CLI. The disaggregated numbers for individual experiments are included in the Source Data Files.

Field-collected samples
Mice were bred, reared and operated under specific-pathogen-free (SPF) conditions with a 12 h/12 h light/dark cycle at 21°C and 50-55% humidity at the animal facilities of Southern Medical University.

Ethics oversight
All experiments were conducted under the Institutional Animal Care and Use Committee of Southern Medical University.

Note that full information on the approval of the study protocol must also be provided in the manuscript.

Plants

Seed stocks
N/A

Novel plant genotypes
N/A

Authentication
N/A
